# Septation of Infectious Hyphae Is Critical for Appressoria Formation and Virulence in the Smut Fungus *Ustilago Maydis*

**DOI:** 10.1371/journal.ppat.1002044

**Published:** 2011-05-19

**Authors:** Johannes Freitag, Daniel Lanver, Christian Böhmer, Kay Oliver Schink, Michael Bölker, Björn Sandrock

**Affiliations:** 1 Department of Biology, Philipps-University Marburg, Marburg, Germany; 2 Max-Planck-Institute for Terrestrial Microbiology, Department of Organismic Interactions, Marburg, Germany; 3 Department of Biochemistry, Institute for Cancer Research, The Norwegian Radium Hospital, Oslo, Norway; Virginia Polytechnic Institute and State University, United States of America

## Abstract

Differentiation of hyphae into specialized infection structures, known as appressoria, is a common feature of plant pathogenic fungi that penetrate the plant cuticle. Appressorium formation in *U. maydis* is triggered by environmental signals but the molecular mechanism of this hyphal differentiation is largely unknown. Infectious hyphae grow on the leaf surface by inserting regularly spaced retraction septa at the distal end of the tip cell leaving empty sections of collapsed hyphae behind. Here we show that formation of retraction septa is critical for appressorium formation and virulence in *U. maydis*. We demonstrate that the diaphanous-related formin Drf1 is necessary for actomyosin ring formation during septation of infectious hyphae. Drf1 acts as an effector of a Cdc42 GTPase signaling module, which also consists of the Cdc42-specific guanine nucleotide exchange factor Don1 and the Ste20-like kinase Don3. Deletion of *drf1, don1* or *don3* abolished formation of retraction septa resulting in reduced virulence. Appressorium formation in these mutants was not completely blocked but infection structures were found only at the tip of short filaments indicating that retraction septa are necessary for appressorium formation in extended infectious hyphae. In addition, appressoria of *drf1* mutants penetrated the plant tissue less frequently.

## Author Summary

Pathogens exhibit various developmental stages during the process of infection and proliferation. The basidiomycete *Ustilago maydis* is a model organism for plant pathogenic fungi. On the plant surface *U. maydis* grows as a cell-cycle arrested filament. Growth of infectious hyphae involves regular formation of retraction septa leaving empty sections behind. The tip cell forms an appressorium and penetrates the cuticle. In this study we identified for the first time a signaling module regulating formation of retraction septa in fungal hyphae. The module consists of the highly conserved small GTPase Cdc42, its activator Don1 and the actin-organizing formin Drf1. After penetration of the plant, cell cycle arrest is released and hyphal septation is resumed in planta but was found to be independent of Cdc42 and Drf1. Thus, during infection Cdc42 signaling and Drf1 coordinate hyphal septation events specifically in infectious hyphae in *U. maydis*. The inability to form retraction septa affects filament elongation and appressorium formation resulting in significantly reduced virulence. We observed a threshold size of the cytoplasm filled tip compartment above which appressorium formation is blocked. These findings highlight that formation of retraction septa, a common feature of filamentous fungi, is an important virulence determinant of *U. maydis*.

## Introduction

Penetration of the plant cuticle is a prerequisite for the establishment of many plant-fungal interactions and involves the formation of specialized infection structures, called appressoria. These structures mediate plant penetration in parasitic as well as in symbiotic fungi [1], [2]. Appressoria are often melanized and contain thickened cell walls, which are necessary to generate the mechanical force to break through the cuticle of the plant epidermis. This process is driven by turgor-derived osmotic pressure and often involves targeted secretion of lytic enzymes [2]–[4]. Induction of appressorium formation is intricately regulated and triggered by chemical signals, hydrophobicity and surface texture [2], [5]. These external stimuli are transmitted through mitogen activated protein kinases and cAMP signaling [6], [7]. The massive reorganisation of the fungal cell observed during differentiation of infection structures is also coupled to cell cycle regulation [2].

**Figure 1:**
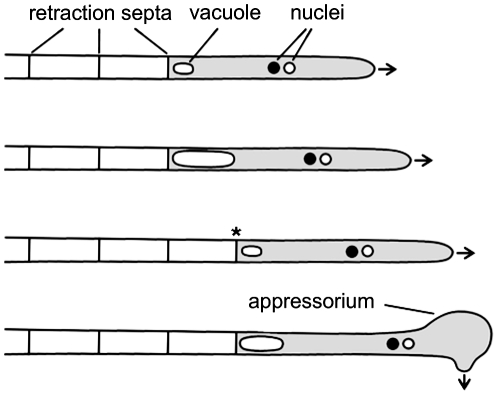
Filamentous growth of *U. maydis* on the plant surface. The dikaryotic filament of *U. maydis* grows by inserting retraction septa at the distal end of the filament. For details see main text. Arrows indicate growth direction; asterisk marks a new assembled retraction septum and the cytoplasm-filled tip compartment is drawn in grey.

The basidiomycetous fungus *Ustilago maydis* infects maize plants and causes smut disease [8], [9]. To infect its host, compatible haploid sporidia fuse and form dikaryotic filaments spreading on the plant surface. These hyphae grow unipolar, do not branch and are arrested in the G2 phase of the cell cycle [8], [10]. At the distal end of the growing filament, regularly spaced retraction septa are inserted, which delimit the cytoplasm-filled tip compartment from empty hyphal sections. Retraction septa are found in many filamentous fungi and ensure high-speed movement without mitosis and de novo generation of cytoplasm (Figure 1) [11]. In *U. maydis* appressorium formation at the tip of filaments is triggered by surface hydrophobicity and cutin monomers [12]. These appressoria are unmelanized and are thought to penetrate the cuticle predominantly by secretion of lytic enzymes, rather than by mechanical force [13]. After penetration cell cycle arrest is released, the fungus proliferates within the plant and induces the formation of tumours, in which diploid teliospores are generated [14].

In *U. maydis* cell fusion is controlled by a pheromone/receptor system encoded by the *a* locus, while subsequent establishment of infectious hyphae depends on an active heterodimer of the homeodomain transcription factors expressed from the multiallelic *b* locus [15]–[17]. It has been shown that *b* is the master regulator of pathogenic development. An effector of *b*, the transcription factor Clp1 is important for the release of the cell cycle arrest when infectious hyphae have invaded the host and mitotic proliferation of hyphae is initiated [18]. For successful plant infection the Rho family GTPase Cdc42 is required [19]. In addition, Cdc42, the Cdc42 specific guanine nucleotide exchange factor (GEF) Don1 and the Ste20-like protein kinase Don3 are required for cell separation of haploid sporidia during budding growth [19]–[22].

Morphogenesis and differentiation of eukaryotic cells involve reorganisation of the actin cytoskeleton. Formin proteins catalyze polymerization of monomeric actin into linear filaments [23], [24] and thus play an important role in the organisation of the actin cytoskeleton [25]. Formins are large multidomain proteins and contain the highly conserved formin homology 2 (FH2) domain at the C-terminus, which is responsible for actin nucleation [26]. Incorporation of monomeric actin is stimulated by the profilin-binding formin homology 1 (FH1) domain [27]. The diaphanous formin was first described in *Drosophila melanogaster* where it is required for cytokinesis [28]. Formins of the diaphanous family are characterized by the presence of an N-terminal Rho family GTPase binding domain and a C-terminal autoregulatory domain [29]. Binding of GTPases of the Rho/Rac-family relieves the autoinhibition between the diaphanous autoregulatory domain (DAD) at the C-terminus and the diaphanous inhibitory domain (DID) resulting in stimulation of actin polymerization [28], [30], [31].

Here we report that in *U. maydis* the diaphanous-related formin Drf1 acts as an effector of Cdc42. Interestingly, *drf1* as well as *don1* and *don3* mutants were unable to form septa in cell cycle arrested infectious hyphae. We observed that these mutants develop infection structures only in short filaments while longer filaments lack appressoria. Therefore, we conclude that in *U. maydis* hyphal septation is critical for appressorium development and plant infection.

## Results

### Mutants lacking *drf1* do not form retraction septa

**Figure 2:**
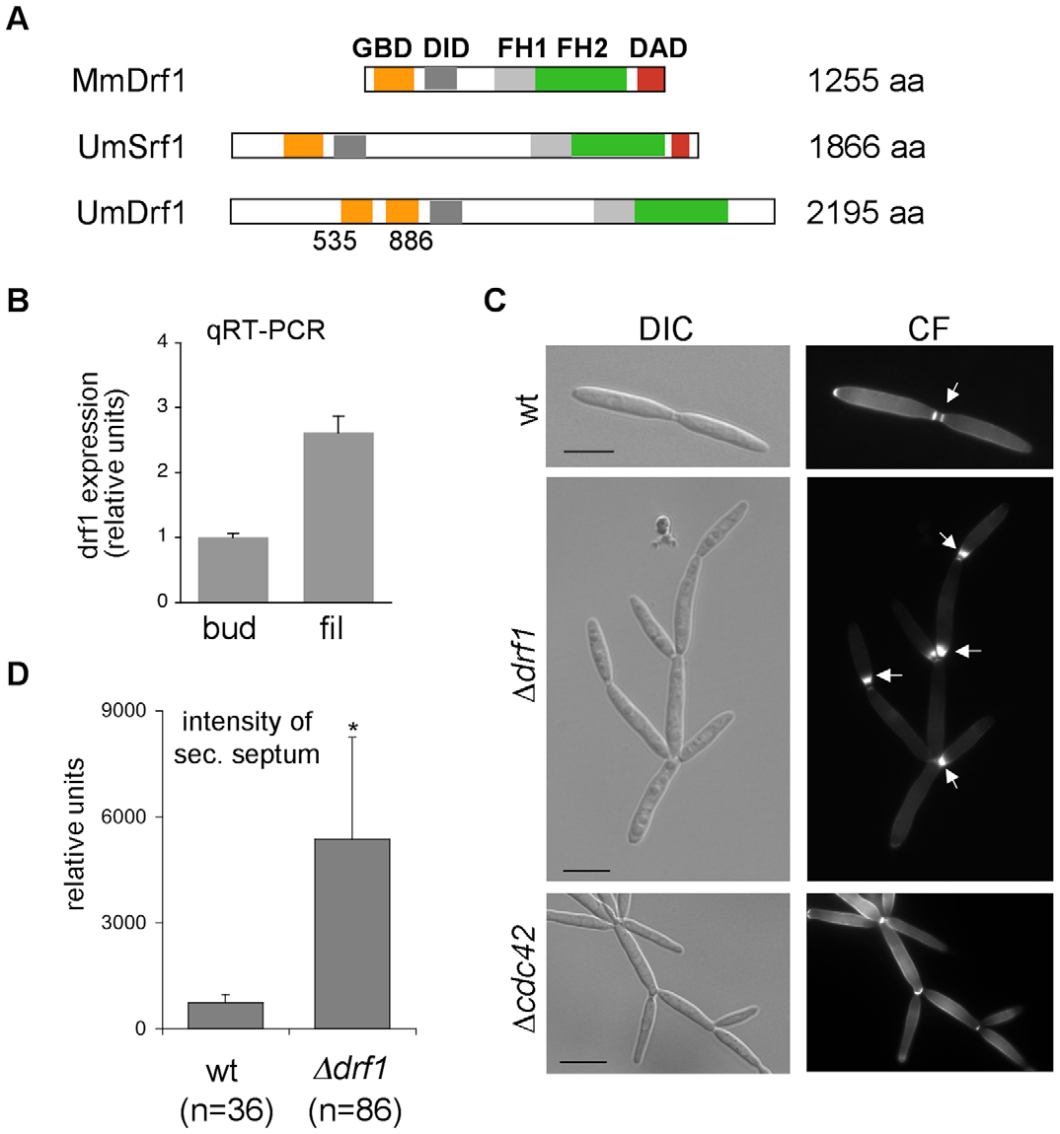
*drf1* expression is induced during filamentous growth and its deletion shows a cell separation defect. A: Domain alignment of MmDrf1 (*Mus musculus*), UmDrf1 and UmSrf1. GBD: GTPase binding domain; FH1: Formin homology domain 1; FH2: Formin homology domain 2; DAD: Diaphanous autoregulatory domain; DID: Diaphanous inhibitory domain. B: Quantitative real time-PCR for *drf1* and *ppi* (um03726) were performed on RNA prepared from budding cells (bud) and filaments (fil). Each column represents the mean ratio *drf1*/*ppi* from three independent experiments. *ppi* served as internal control. C: wt, Δ*drf1* or Δ*cdc42* mutant cells were grown in rich media and stained with calcofluor white (CF). Abnormal chitin depositions are labelled with arrows. D: The secondary septa of wt and Δ*drf1* mutant cells from logarithmic cultures were stained with calcofluor white and the intensities were measured using ImageJ. *: p<0.05, when compared to the wt strain. (Scale bars: 10 µm).

*U. maydis* contains two members of the formin family, the SepA-related Srf1 and the diaphanous-related formin Drf1 (Figure 2A). Both proteins contain the characteristic FH1, FH2 and DID domains. Interestingly, in Drf1 the GTPase binding domain (GBD) is split and the protein lacks the conserved DAD domain (Figure 2A). Genome wide expression analysis revealed that expression of *drf1* but not of *srf1* is significantly induced during *b*-dependent filament formation [18]. This suggests a role for this formin during pathogenic development, which in *U. maydis* is controlled by the heterodimeric bE/bW homeodomain transcription factor. We confirmed the filament-specific induction of *drf1* expression using qPCR (Figure 2B). For this purpose we used the strain AB31, in which filamentation can be induced by arabinose dependent transcription of *bW*/*bE* [32].

To investigate the cellular function of Drf1 we deleted *drf1* in the *U. maydis* wild type strain Bub8. Cells lacking *drf1* were viable and exhibited normal cell shape. However, Δ*drf1* cells formed clusters of up to 20 cells (Figure 2C), indicating that cell separation between mother and daughter cells is affected in the absence of Drf1. Normally budding cells form two distinct septa at the site of cell separation. The primary septum is sufficient to separate the cytoplasm of mother and daughter cells and is formed at the mother side of the bud neck. The secondary septum is needed to completely separate cells from each other and is formed at the daughter side of the bud neck [22]. The secondary septum was not formed in *drf1* mutants, which could account for the cell separation defect. Interestingly, *drf1* mutants deposited massive amounts of cell wall material at the site where the secondary septum is usually formed and this most likely leads to a delayed cell separation (Figure 2C). We also quantified these accumulations and found that their intensity is about six times increased in comparison with secondary septa of wild type budding cells (Figure 2D). These accumulations of cell wall material were not detected in *cdc42* mutants, which also display a cell separation defect (Figure 2C). This indicates additional functions of Cdc42 during cell separation, maybe in chitin deposition [19].

**Figure 3:**
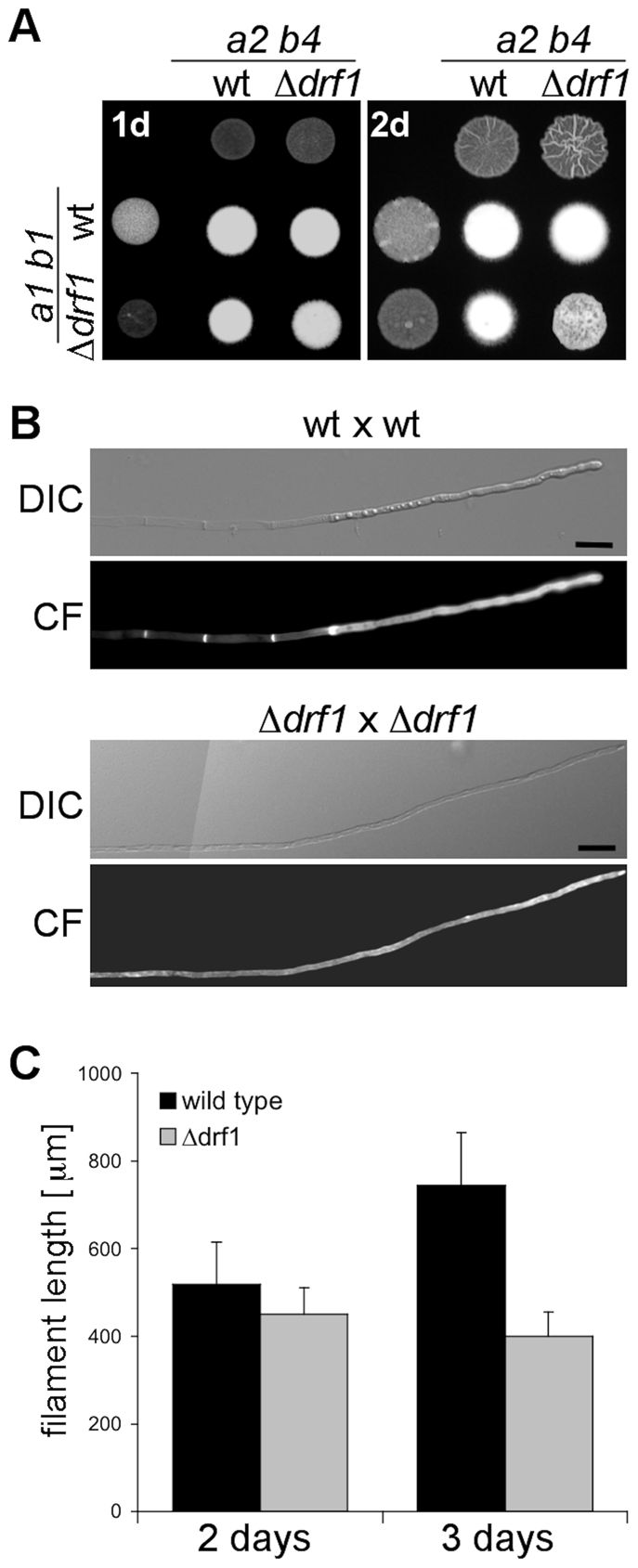
Filaments of *drf1* mutants do not form retraction septa. A: The indicated haploid strains were spotted either alone or in combination on charcoal-containing medium. White colonies are indicative for the formation of aerial hyphae. Filamentous growth was analyzed one (1d) and two (2d) days after spotting the respective strains. B: Two-day-old filaments formed by the indicated crossings were stained with calcofluor white (CF; lower panel). The respective brightfield image is shown (upper panel). Filaments of *drf1* deletion strains lack retraction septa. To depict more than 180 µm of the growing filament it was necessary to combine two images (Scale bars: 15 µm). C: Quantification of filament length. Using the same strains as in B, filament length was calculated using ImageJ. For each strain and time point 30 filaments were analyzed. Error bars indicate standard deviation.

To investigate the role of Drf1 during mating and filament formation we performed mating assays on charcoal-containing agar plates. In this assay, fusion of compatible haploid cells and the subsequent switch to hyphal growth results in the formation of a white mycelium [33]. If compared to wild type cells, *drf1* mutants exhibited significantly reduced mycelium formation on charcoal agar (Figure 3A). Interestingly, this difference became obvious only after two days while after 24 hours a comparable number of short hyphae was visible on the surface of colonies (Figure 3A). This suggests that *drf1* mutants are not affected in cell fusion during mating but exhibit a defect in hyphal development.

Microscopic analysis of calcofluor white stained filaments revealed that *drf1* mutants lack hyphal septa in all filament-inducing conditions tested during this study (Figures 3B, S1). By contrast wild type filaments display regularly spaced retraction septa at the rear end of the growing tip compartment (Figures 3B, S1) [11]. In wild type cells, the sections between these hyphal septa were empty and the cytoplasm of the dikaryotic cell was confined to the apical compartment (Figures 1, 3B) [34]. In *drf1* mutants, the lack of hyphal septation resulted in a marked elongation of the apical cytoplasmic compartment. Since *drf1* mutants displayed reduced mycelium formation in the plate-mating assay (Figure 3A) we asked whether deletion of *drf1* affects the growth rate of filaments. Two days after fusion, filaments of wild type strains and *drf1* mutants were indistinguishable in length and reached approximately 400 µm. After three days Δ*drf1* filaments had stopped elongation while wild type filaments continued to elongate (Figure 3C). This observation suggests that *U. maydis* hyphae require distal retraction septa formation to cover long distances.

We wondered whether depletion of Drf1 influences polar exocytosis as it has been shown for AgBni1 in *Ashbya gossypii* [35]. This could possibly cause the growth defect of *b*-induced filaments in absence of Drf1. Therefore we fused the Rab GTPase Sec4 to GFP and analyzed the localization of the fusion protein in AB31 and AB31Δ*drf1* filaments. Remarkably, in *U. maydis* Sec4 did not show predominant tip localization as it has been observed in *A. gossypii* [35], but was found on moving vesicles throughout the filament (Figure S2). Longer filaments derived from *drf1* mutants showed a reduced density of these vesicles presumably due to the enlarged cytoplasmic volume in the absence of retraction septa (Figure S2).

### The ability to form a secondary septum appears to be prerequisite for hyphal septation

**Figure 4:**
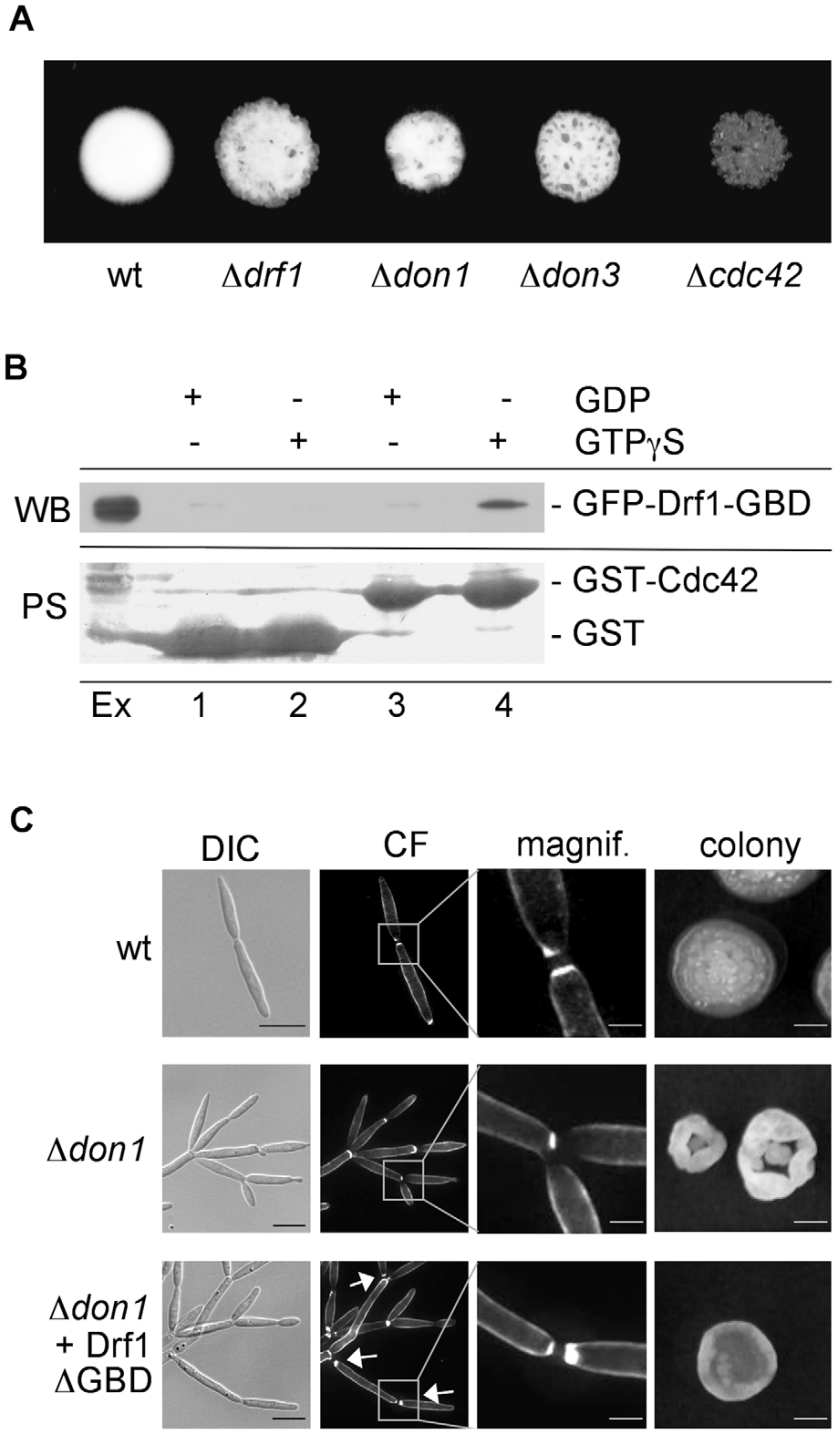
Drf1 is an effector of Cdc42. A: Compatible mixtures of the indicated haploid strains were spotted on charcoal-containing medium. Plates were incubated for two days. B: Pull-down experiment of GFP-Drf1-GBD: GST (lanes 1 and 2) or GST-Cdc42 (lanes 3 and 4) loaded with GDP (lanes 1 and 3) or GTPgS (lanes 2 and 4) were loaded with *U. maydis* protein extract containing GFP-Drf1-GBD (Ex). After pull-down immunoblotting (WB) was performed with anti-GFP antibody. Blots were stained with PoinceauS (PS) as loading control. GTP-bound Cdc42 could interact with the GBD of Drf1. C: Constitutive active Drf1ΔGBD was able to partially suppress the *don1* deletion phenotype. Wt, Δ*don1* and Δ*don1*+pETEF-Drf1ΔGBD were stained with calcofluor white (CF) (central columns). Arrows indicate secondary septum formation. For the colony morphology images (right column) single cells were grown for three days on YEPS with 1.3% Agar. (Scale bars: 10 µm for the left column, 2 µm for the magnifications and 1 mm for the colonies).

*drf1* mutants not only lack hyphal septa but also display a pronounced cell separation defect during budding (see above), which resembles the phenotype of *U. maydis* strains deleted for *don1*, *don3* and *cdc42*. These genes constitute a Cdc42 GTPase signaling network that regulates secondary septum formation during cytokinesis [19], [22], [36]. *don1*, *don3* and *cdc42* mutants are unable to separate daughter cells after mitosis, but in contrast to *drf1* mutants accumulations of cell wall material are not detectable [19], [22]. We tested whether Cdc42 signaling is also required for hyphal septation. In plate-mating assays on charcoal plates, Δ*don1* and Δ*don3* mutants formed colonies with reduced mycelium comparable to Δ*drf1* mutant colonies (Figure 4A). Since Δ*cdc42* mutants are already affected in cell fusion [19], colonies on charcoal plates showed only scattered mycelia (Figure 4A). Microscopic analysis of Δ*don1* and Δ*don3* filaments revealed that these mutants were also defective in hyphal septation (Figure S3) suggesting that this process involves the same regulatory network as secondary septum formation during budding.

### Drf1 acts as an effector of Cdc42

The genetic interaction between Cdc42 signaling and Drf1 during hyphal septation suggests that the N-terminal GTPase binding domain (GBD) of Drf1 (Figure 2A) may interact with Cdc42. We used GST-Cdc42 to perform pull-down experiments with *U. maydis* whole cell extract. GFP-GBD_Drf1_ was able to interact with Cdc42 in its active GTP-bound form but not in its inactive GDP-bound form (Figure 4B).

Deletion of the GBD can result in constitutive active formin variants [37]. When drf1ΔGBD was introduced into a Δ*drf1* strain, transformants displayed normal cell separation and retraction septum formation during filamentous growth. This observation shows that Drf1ΔGBD is fully functional (Figures S4B, C). Furthermore, Drf1ΔGBD partially suppressed the cell separation defect of *don1* mutants (Figure 4C). We were able to detect secondary septa in *don1* mutants, which expressed Drf1ΔGBD. These septa were never detected in Δ*don1* cell clusters (Figure 4C). Moreover, the colony morphology of *don1* mutants appeared to be similar to wild type colony morphology when Drf1ΔGBD was expressed (Figure 4C). This indicates that the truncated protein is constitutively active. Drf1ΔGBD was unable to rescue the cell separation defect of *cdc42* mutants (Figure S5), implying additional functions of Cdc42 during cell separation. These functions might be regulated by other Cdc42-specific GEFs, which already have been identified in *U. maydis* (Britta Tillmann, unpublished data). Altogether we assume that in *U. maydis* the diaphanous-related formin Drf1 acts as an effector of Cdc42 during cell separation and during hyphal septation on the plant surface.

### *drf1* mutants are unable to form contractile actomyosin rings in filaments

**Figure 5:**
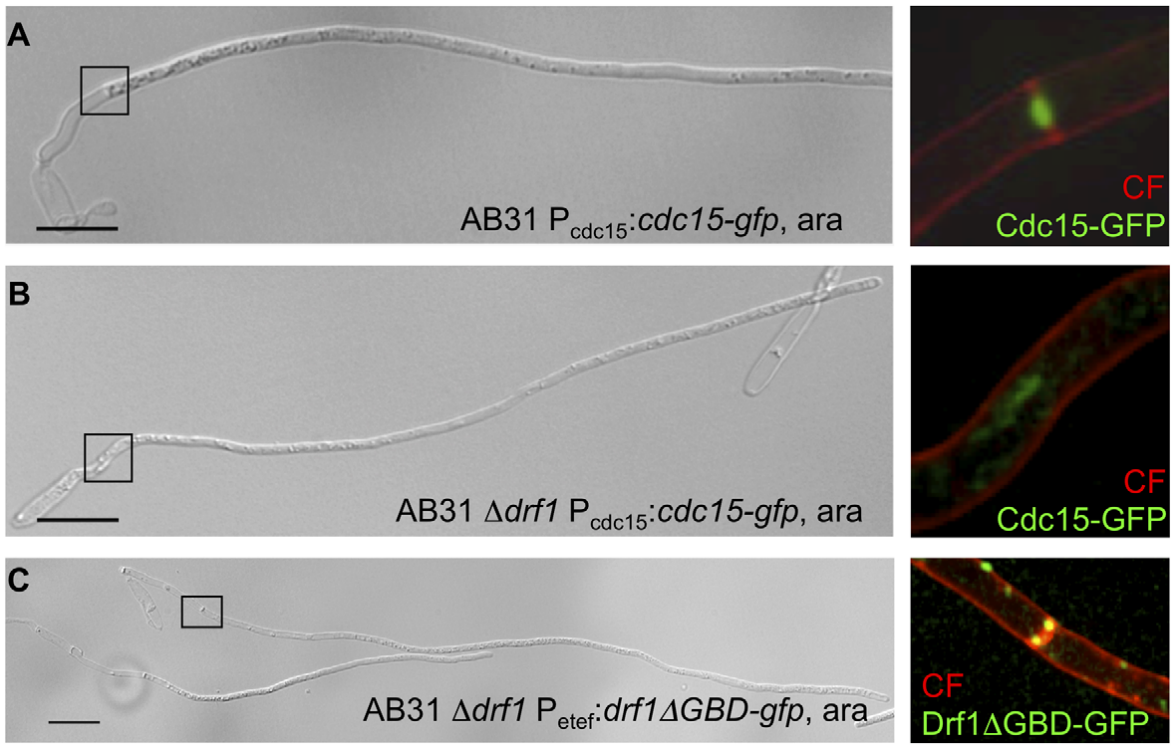
Hyphal septation depends on CAR formation. A: CAR formation in wt filaments is shown by the actomyosin ring marker Cdc15-GFP (green) and co-staining with calcofluor white (red). The magnification shows the formation of the first distal septum (red) while the actomyosin ring started to constrict (green). B: The same settings were used for the AB31Δ*drf1* mutant expressing Cdc15-GFP. The region of the bud neck is magnified. C: The constitutive active variant Drf1ΔGBD-GFP (green) was expressed in AB31Δ*drf1* and localized in filaments at the site of restored hyphal septation (red). (Scale bars: 15 µm).

During budding growth *U. maydis* forms two septa to facilitate separation of mother and daughter cells. Both, the primary and secondary septation event in *U. maydis* involve the formation of a contractile actomyosin ring (CAR) [36], [38]. It has been shown that the Cdc42-GEF Don1 and the Ste20-like protein kinase Don3 are required to trigger CAR formation specifically during the secondary septation event but not during primary septation [38]. To visualize CAR formation during hyphal septation we expressed the F-BAR domain protein Cdc15-GFP in strain AB31. Cdc15 is an integral part of the CAR during both primary and secondary septum formation in *U. maydis* [36]. When filament formation of AB31 was induced with arabinose a ring-like accumulation of Cdc15-GFP fluorescence was detected during retraction septa formation (Figure 5A). In AB31Δ*drf1* no ring-like accumulation of Cdc15-GFP could be observed (Figure 5B). CAR formation was also abolished in hyphae of *don1* and *don3* mutants (Figure S6, protocol S1). Furthermore *drf1* mutants failed to assemble the CAR at the site of secondary septation in haploid cells (Figure S7). Together, these data suggest that hyphal septation involves assembly of a CAR, which requires beside the formin Drf1 also the Cdc42/Don1/Don3 signaling network.

To further analyze the role of Drf1 during CAR-formation we created GFP fusion proteins of Drf1. Unfortunately, neither N-terminal nor C-terminal GFP-tagged Drf1 was able to rescue the Δ*drf1* phenotype (data not shown). Thus, we fused *gfp* to the C-terminus of the putative dominant active drf1ΔGBD, expressed this fusion protein in *drf1* mutants and investigated the localization of Drf1ΔGBD-GFP in budding cells and in *b*-induced filaments, respectively. We found Drf1ΔGBD-GFP to be able to complement the phenotype of *drf1* deletions, indicating that the protein is functional. In budding cells, Drf1ΔGBD-GFP localized at punctuated structures. Furthermore, during separation of budding cells Drf1ΔGBD-GFP clearly co-localized with the secondary septum at the daughter site (Figure S4D). In *b*-induced filaments Drf1ΔGBD-GFP localized at the site where retraction septa form (Figure 5C). These results confirm the specific function of Drf1 during both, secondary septa formation in budding cells and retraction septa formation during filamentous growth.

### Formation of septin rings depends on Drf1

It has been shown previously that formation of the secondary septum in *U. maydis* involves the formation of a septin collar at the site of secondary septum formation [38]. This collar is subsequently disassembled followed by CAR-formation. After CAR formation a septin ring assembles de novo and cell separation can be carried out [38]. We wondered whether septin organistion is impaired in *drf1* mutants and consequently performed localization studies with a RFP-tagged septin (Cdc10-RFP). We found that in budding Δ*drf1* cells a septin collar structure formed at the expected site of the secondary septum (Figure S8B). We never detected the transition to septin rings (Figures S8A, B). The assembly of septin rings seems to depend on Drf1. We also studied the localization of Cdc10-RFP in *b*-induced filaments. Cdc10-RFP was found to co-localize with calcofluor white stained retraction septa in wild type cells while in *drf1* mutants no ring structures were observed (Figure S8C, D). Moreover, Cdc10-RFP localized in dispersed patches throughout the filament as described previously [39]. This type of localization was not changed in *drf1* mutants.

### *U. maydis drf1* mutants display reduced virulence

To examine, if the inability to form retraction septa affects pathogenic development of *U. maydis,* we determined the virulence of *drf1* mutants. Wild type and *drf1* mutant cells were used to infect seven-day-old maize seedlings. While injection of wild type cells triggered tumour formation in 96% of infected plants (14 days post infection, dpi), plants inoculated with *drf1* mutant cells displayed significant attenuated symptoms and induced tumours in only 16% of infected plants (Table 1). Virulence was completely restored when the open reading frame of *drf1* under control of the constitutive *otef* promoter was reintroduced into the mutants (Table 1) [40]. In the complemented strains, normal cell separation and hyphal septation have also been restored (Figures S4A, C).

**Table 1:** Pathogenicity assay.

	plants	tumours
*a1 b1* × *a2 b4*	45	42 (93%)
Δ*drf1* × Δ*drf1*	126	20 (16%)
Δ*drf1* × Δ*drf1*+Drf1	75	68 (88%)

### Retraction septa are critical for virulence since they are required for appressorium formation in longer filaments

**Figure 6:**
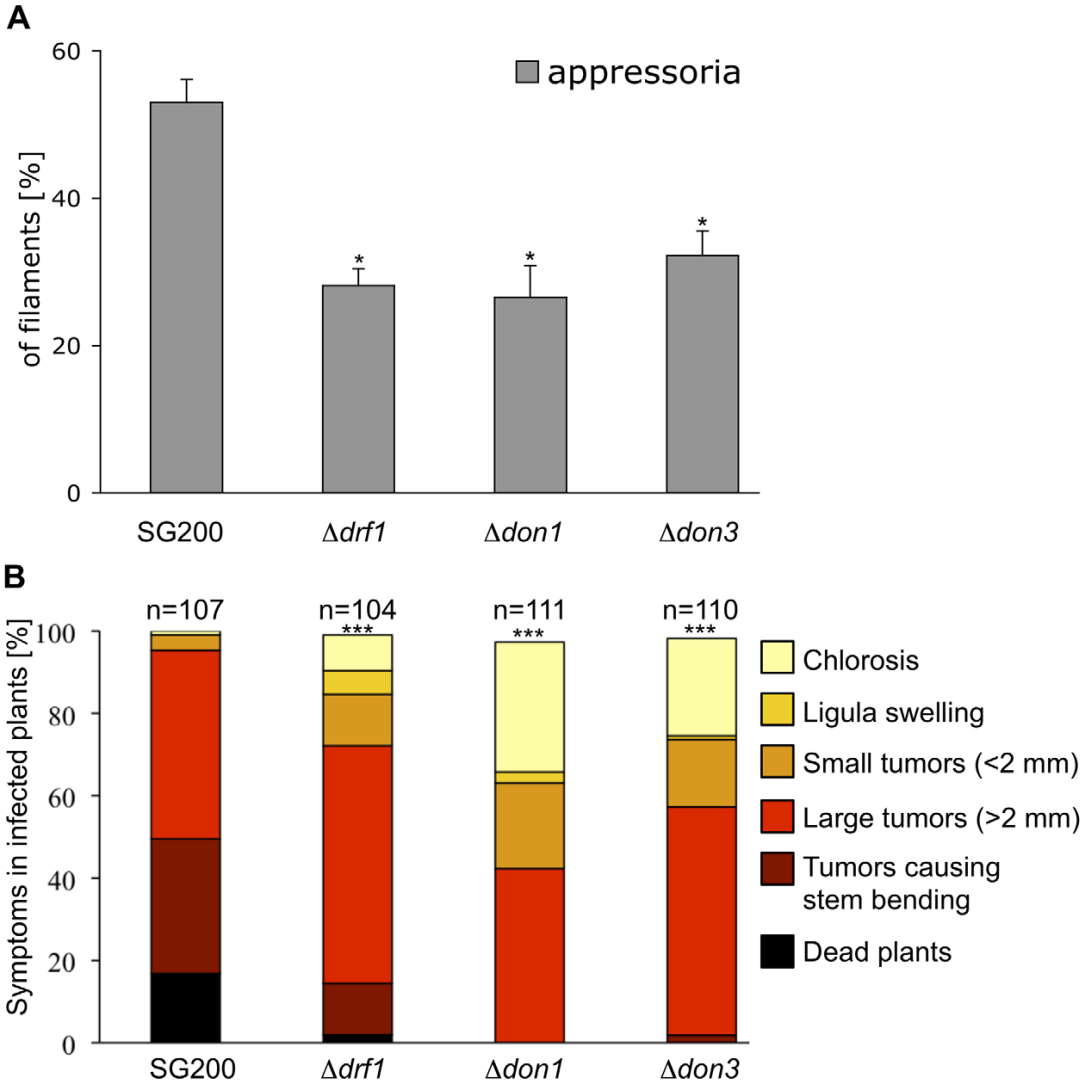
Appressoria formation and virulence are reduced in the *drf1, don1* and *don3* deletion strains. A: The indicated strains were injected into maize seedlings. One day after infection appressoria were quantified on the leaf surfaces using the appressorium specific marker AM1 (n>200). *: p<0.05, when compared to the SG200AM1 strain. The experiment was conducted in three biological replicates. Error bars indicate standard deviations. B: Disease symptoms caused by the solopathogenic SG200AM1 strain and its derivative *drf1*, *don1* and *don3* deletion strains (indicated below each column) were scored 12 days after infection. Symptoms were grouped into colour-coded categories according to Kämper et al. [9] depicted on the right. Average values of three independent experiments were expressed as percentage of the total number (n) of infected plants. ***: p<0.0001 using the Wilcoxon ranked sum test, when compared to the SG200AM1 strain.

Next we asked whether the hyphal septation defect of *drf1* mutants is responsible for reduced virulence. Since virulence defects may result from reduced penetration or from reduced growth within the plant, we analyzed the infection process. The first step during infection is the formation of appressoria. Because appressoria in *U. maydis* are difficult to detect, we used the solopathogenic strain SG200AM1, which expresses an appressorium specific GFP reporter [9], [12]. *drf1*, *don1* or *don3* were deleted in SG200AM1 and appressorium formation was investigated on the plant surface. We found that all three mutants differentiate appressorial structures (Figure S9). However, quantification of these infection structures revealed a significant reduction of appressorium development in Δ*drf1*, Δ*don1* and Δ*don3* strains (Figure 6A). Furthermore, we determined virulence of the SG200AM1 derivative *don1*, *don3* and *drf1* mutants. The progenitor strain SG200AM1 induced tumours in 95% of the plants. SG200AM1Δ*don1*, SG200AM1Δ*don3* and SG200AM1Δ*drf1* showed significantly reduced virulence (Figure 6B). In particular the severest symptoms, i.e. dead plants were observed in only 2% of SG200AM1Δ*drf1* infected plants, while 19% dead plants were scored for SG200AM1 infections (Figure 6B). This reduction of virulence supports our notion that Drf1, Don1 and Don3 play an important role in the initial phase of pathogenic development.

**Figure 7:**
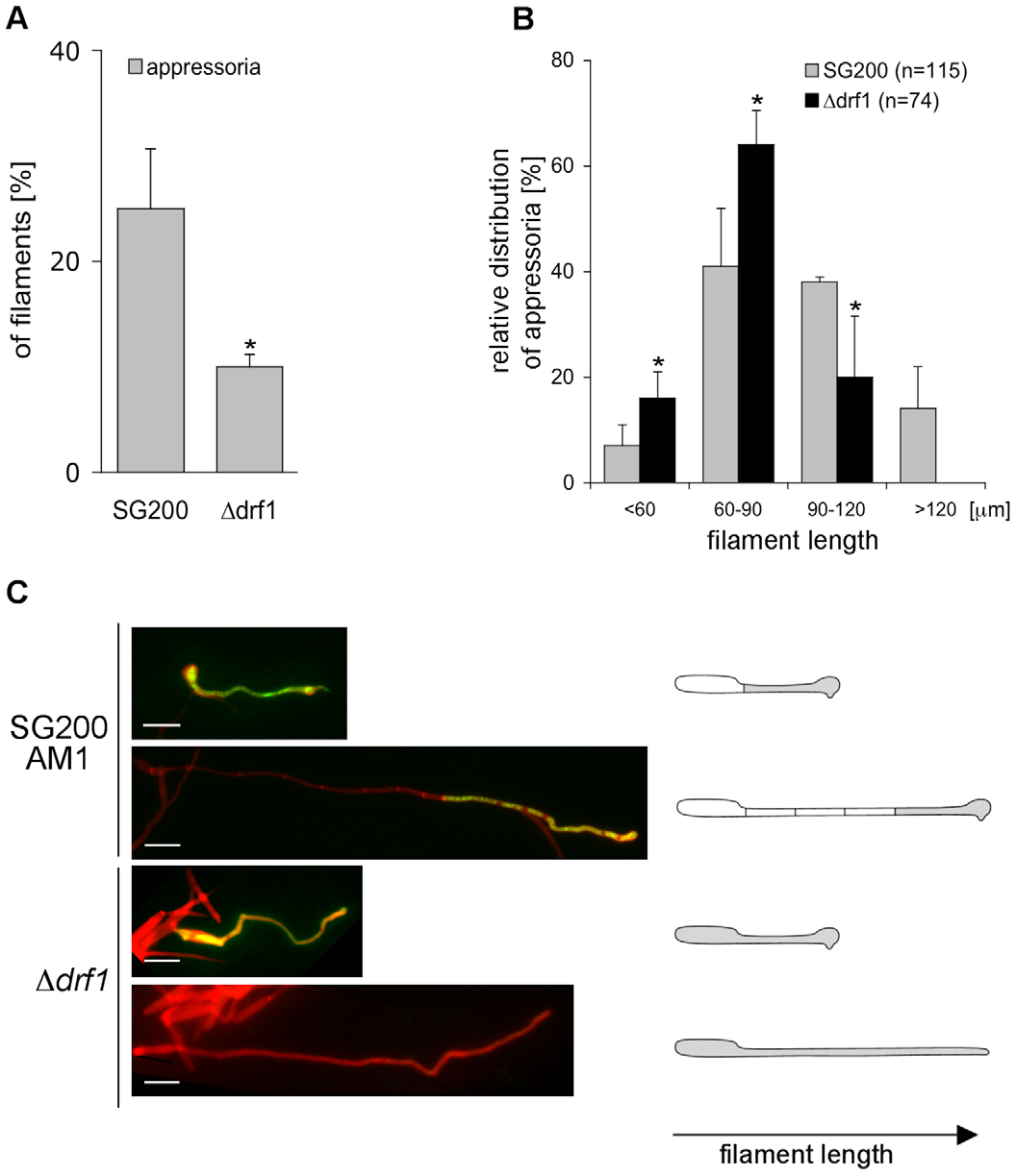
*drf1* mutants are affected in appressoria formation in long filaments. A: Appressoria formed by the solopathogenic SG200AM1 strain and its derivative *drf1* deletion strain were quantified 20 hours after spraying cells on Parafilm in the presence of 16-hydroxyhexadecanoic acid. *: p<0.05, when compared to the SG200AM1 strain. B: Distribution of appressoria formed by filaments of different length was compared between SG200AM1 and SG200AM1Δ*drf1*. *: p<0.05, when compared to the SG200AM1 strain. Errorbars indicate standard deviation. C: Filaments were visualised using calcofluor white (red). Appressorium formation is indicated by expression of the AM1 marker (green). When filaments of *drf1* mutants exceeded a certain length appressoria did not develop.

Appressorium formation can also be induced on an artificial hydrophobic surface in the presence of long-chain hydroxy fatty acids [12]. Under these in vitro conditions appressorium formation of *drf1* mutants was again significantly reduced if compared to the progenitor strain SG200AM1 (Figure 7A). The appressorial structures of SG200AM1 and SG200AM1Δ*drf1* were indistinguishable in morphology and had a similar diameter (Figure S11). However, we noticed that appressorium formation in Δ*drf1* mutants occurred predominantly at the tip of short filaments (60–90 µm) while in wild type filaments appressoria were formed also at the tip of longer filaments (Figures 7B, C and S10). Remarkably, filaments of *drf1* mutants, which exceeded 120 µm were unable to form appressoria (Figure 7B). Together these data suggest that formation of retraction septa in *U. maydis* is not required for appressorium formation in short infectious hyphae while appressorium differentiation in longer filaments (>120 µm) requires hyphal septation. We conclude that a limited, cytoplasm-filled tip compartment is critical for appressorisum formation.

### Appressoria derived from *drf1* mutants penetrate the host cuticle less frequently

**Figure 8:**
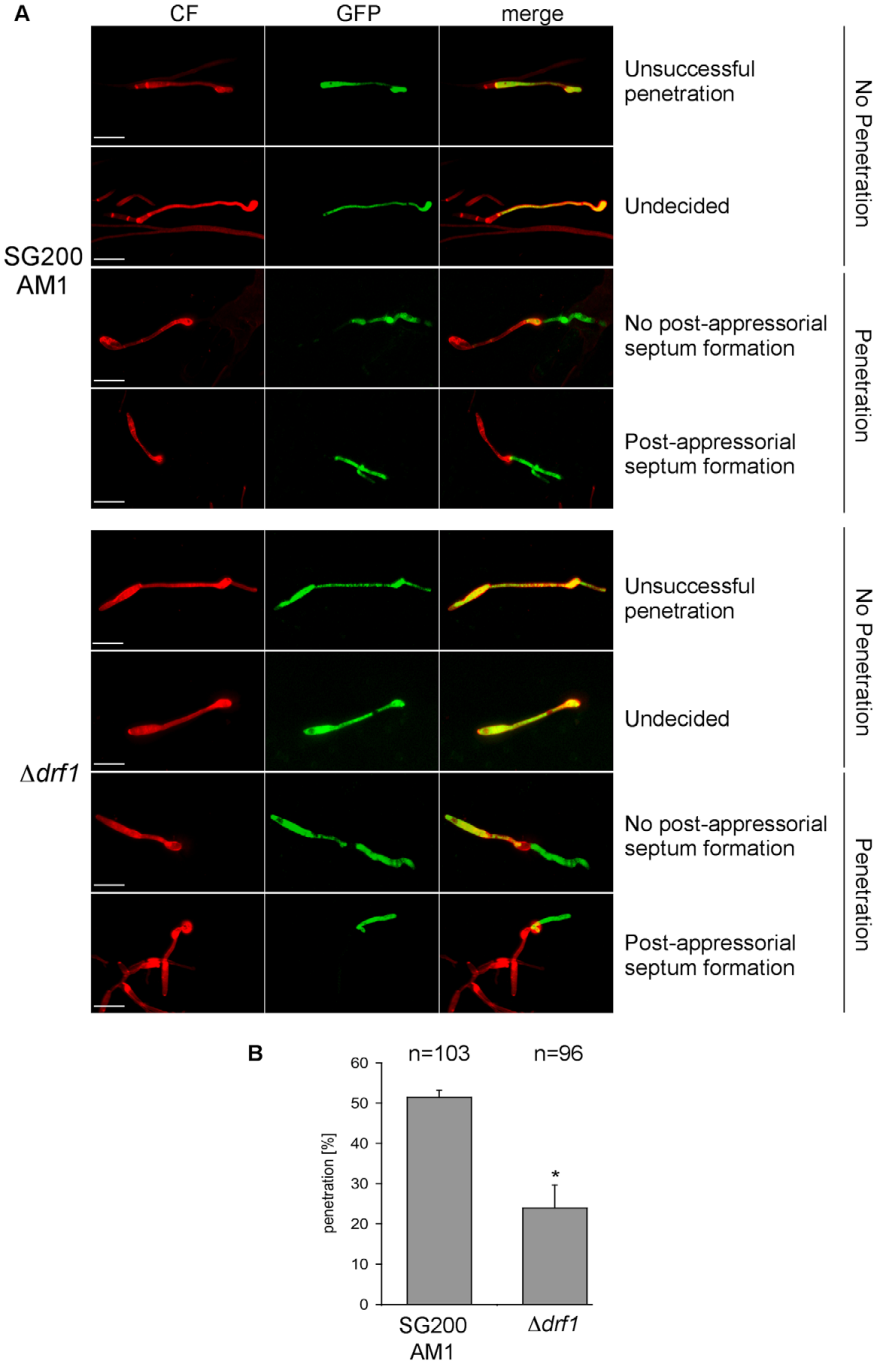
Penetration efficiency from appressoria of *drf1* mutants is reduced. A: Confocal projections of colonized leaf tissue 18 hours after infection with the indicated strains. Fungal material on the leaf surface was stained with calcofluor white (red) and expression of the AM1 marker was monitored (green). “Unsuccessful penetration” describes appressoria that continued growth on the plant surface. “Undecided” describes appressoria, which have just been formed. Penetration events are divided in “no post-appressorial septum formation”, which is characterized by GFP-fluorescence in penetrating hyphae as well as in hyphal parts on the plant surface and “post-appressorial septum formation”, where GFP-fluorescence was exclusively found in penetrating hyphae. (Scale bars: 20 µm) B: The penetration efficiency was calculated by analyzing the indicated number of filaments from three independent experiments. *: p<0.05, when compared to the wt strain. Errorbars indicate standard deviation.

So far we have analyzed the connection between appressorium differentiation and retraction septum formation. We also wondered if the ability to penetrate the plant cuticle was impaired in *drf1* mutants. We therefore compared the penetration frequencies between appressoria derived by SG200AM1 and SG200AM1Δ*drf1*. Using confocal microscopy 18 h after inoculation of maize plants, appressoria were found either to penetrate the plant surface, to continue growth on the leaf surface or to be in-between these decisions (Figure 8A). Appressoria derived by SG200AM1Δ*drf1* penetrated the plant cuticle significantly less if compared to appressoria derived by SG200AM1 (Figure 8B). Only about 25% of appressoria successfully invaded the host in *drf1* mutants while about 50% of SG200AM1 appressoria reached the host tissue (Figure 8B). This indicates that the penetration ability of differentiated appressoria is also affected in the absence of retraction septa. Interestingly, *drf1* mutants were found to be capable of forming septa after having penetrated the plant surface (Figure 8A), indicating that septation events inside the plant are independent of Drf1.

### Drf1/Don1/Don3 are dispensable for cytokinesis in the host

**Figure 9:**
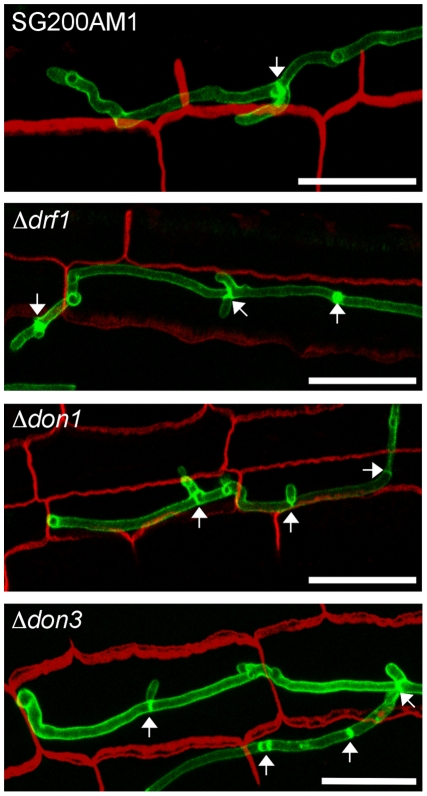
Septum formation of *drf1*, *don1* and *don3* deletion mutants in planta. Confocal projections of colonized leaf tissue three days after infection with the indicated strains. Fungal hyphae were stained by WGA-AF488 (green). Plant tissue was stained by propidium iodide (red). The formation of septa in intracellular growing hyphae is indicated by arrows. (Scale bars: 25 µm).

After penetration G2 arrest is released and hyphae grow intracellularly. Thereby mitotic septa are formed to separate nuclei [41]. Therefore we investigated the role of the Don1/Cdc42/Drf1/Don3 signaling network during the biotrophic phase of *U. maydis*. We observed that hyphal growth of *drf1*, *don1* and *don3* mutants in planta was indistinguishable from wild type hyphae with respect to septum formation (Figure 9). Overall, our results demonstrate that *U. maydis* produces two distinct classes of septa. The primary septum of haploid budding cells, as well as septa formed during growth in the plant are coupled to mitosis and do not require the Cdc42 signaling module. By contrast, the secondary septum of haploid budding cells and septa of infectious hyphae are independent of mitosis and require the Cdc42 signaling module.

## Discussion

In this study we could show that formation of retraction septa in *U. maydis* infectious hyphae depends on the diaphanous-related formin Drf1. Drf1 acts as an effector of the small GTPase Cdc42 and is together with the Cdc42-GEF Don1 and the Ste20-like kinase Don3 required for the formation of a contractile actomyosin ring during septation. Remarkably, non-septated infectious hyphae, that have exceeded a certain length, are unable to form appressoria, while short hyphae lacking retraction septa form appressoria. This led to a significant reduction in the formation of functional infection structures and thus explains the attenuated virulence of *drf1*, *don1* and *don3* mutants.

Formins are major players in actin organisation. The genome of *U. maydis* encodes along with the diaphanous-related formin Drf1, a second formin, Srf1, which belongs to the subfamily of SepA-related formins (MIPS *U. maydis* data base) [29]. While *srf1* is an essential gene (B. Sandrock, unpublished data), we found that *drf1* deletion mutants in *U. maydis* display delayed cell separation during budding growth as well as a complete block in hyphal septation. In fungi, diaphanous-related formins have been characterized so far only in ascomycetes, where they regulate actin polymerisation during polar growth and cytokinesis [42]–[44]. *S. cerevisiae* and *A. gossypii* contain two diaphanous-related formins, which are functionally redundant and synthetically lethal [35], [45], [46].

We observed that *U. maydis drf1* mutants were unable to form a contractile actomyosin ring (CAR) during growth of G2 arrested infectious hyphae. We have previously shown that in budding cells Cdc42, its activator Don1 and the protein kinase Don3 are required for CAR formation specifically during secondary septum formation but not during the primary septation event [36]. Here we found that in budding cells Drf1 is essential for CAR formation during secondary septation and also for the subsequent assembly of septin rings. This confirmed that CAR formation is a prerequisite for septin ring assembly during secondary septum formation in budding *U. maydis* cells as it has been suggested previously [38]. Drf1 might fulfil similar functions during CAR formation in *U. maydis* as the formin Cdc12p in fission yeast [47]. It is well established that Cdc12p in *S. pombe* is necessary for CAR formation and also for the regulation of cytokinesis [48]–[50]. In *U. maydis don1/cdc42/don3* mutants showed a defect in hyphal CAR formation and thus lack retraction septa. Therefore we assume that the same cellular machinery operates both in formation of the secondary septum and the retraction septum. The fact that *drf1* mutants are not affected in CAR formation during cytokinesis suggests that Srf1 could be responsible for CAR formation during the primary septation event in budding cells. In higher eukaryotes, diaphanous-related formins interact with diverse GTPases such as RhoA, RhoD and Cdc42 and regulate formation of actin stress fibres, endosome dynamics and cytokinesis [51]. In *Dictyostelium discoideum* dDia2 binds to Rac1 and controls dynamics of filopodia [52]. We demonstrate here that Drf1 binds Cdc42 in its active GTP-bound form, but not in its inactive status. Together with the finding that a constitutive active variant of Drf1 is able to suppress the cell separation defect of *don1* mutants we conclude that in *U. maydis* Drf1 acts as an effector of Cdc42. Retraction septa are also found in other fungi where similar signaling events and mechanisms might operate [53], [54].

Prior to plant invasion *U. maydis* forms infectious hyphae. During this developmental stage the cell cycle is arrested and the filaments grow only at their tips. At the distal end of the cytoplasm-filled tip cell regularly spaced septa are laid down, leaving empty compartments of the collapsed hyphae behind [11], [34]. By this mechanism, the cytoplasm-filled tip compartment is maintained at a constant length of about 150 µm [11]. In *drf1* mutants the cytoplasmic tip compartment was significantly stretched reaching up to 400 µm. Since these mutants stopped hyphal elongation, the super-sized length of the cytoplasmic compartment appears to be limiting for further growth. Hence, the ability to form retraction septa is a prerequisite for the rapid elongation of infectious hyphae, which continue to grow over several days and can reach up to 2000 µm within 24 h [11]. We found that appressorium development in SG200Δ*drf1* strains was restricted to short hyphae up to 120 µm. By contrast, corresponding wild type filaments were able to differentiate appressoria independently of filament length. Consequently, *drf1* mutants were reduced in appressorium formation and virulence. We observed that virulence of mixtures of compatible haploid Δ*drf1* strains was more attenuated than virulence of solopathogenic *drf1* mutants. Since compatible haploid cells have to form conjugation tubes and to fuse prior to infection, this suggests that the additional length of conjugation tubes may further restrict the ability of Δ*drf1* strains to form infection structures.

All mutants affected in formation of retraction septa characterized in this study were reduced in appressorium formation. This raises the question how appressorium formation is affected in these mutants. The exact mechanism, by which *U. maydis* form appressoria is currently unknown [13]. The rice pathogen *M. grisea* forms a highly melanized appressorium, which accumulates massive amounts of glycerol to generate enormous turgor pressure to penetrate the leaf cuticle mainly by mechanical force [2], [3], [55], [56]. Our results open the possibility that for appressoria formation of *U. maydis* turgor pressure might be of importance. The distal septum could act as mechanical support to generate sufficient turgor pressure during appressorium formation. Alternatively, the extended volume of the cytoplasmic tip cell results in dilution of osmotically active substances and hence insufficient turgor pressure is generated to differentiate appressoria. This is supported by the finding that appressoria derived from *drf1* mutants frequently fail to penetrate the plant surface (Figure 8A). It has been suggested that secretion of lytic enzymes is necessary for plant penetration by *U. maydis* [13], [57]. Because we observed a reduction in the density of exocytotic vesicles in *drf1* mutants it is possible that secretion of lytic enzymes is reduced. In *M. grisea* nuclear migration into the appressorium and subsequent autophagic programmed cell death of the conidial mother cell is a prerequisite for successful penetration [58], [59]. Interestingly, *M. oryzae sep1* mutants form irregular septa during germ tube and appressorium formation and are also unable to differentiate functional appressoria [60]. While *M. grisea* appressorium formation displays hallmarks of a strict developmental program and occurs only in the close vicinity of the conidium, *U. maydis* is able to cover long distances before initiation of infection structures. Under natural conditions efficient infection might require growth or movement over long distances to reach young meristematic tissue, which is prerequisite for *U. maydis* to enter the plant [61]. The fact that non-septated filaments of *U. maydis* stopped extension upon a length of approximately 400 µm and were unable to differentiate appressoria when exceeded a length of 120 µm emphasizes the importance of septa for virulence of *U. maydis*. In wild type hyphae the cytoplasm-filled tip compartment can reach up to 150 µm and appressorium differentiation still remains unaffected. Interestingly, *drf1*, *don1* or *don3* deletion strains fail to form appressoria when exceeded this particular length. Thus, 150 µm seems to be the critical length for *U. maydis* filaments to differentiate appressoria.

**Figure 10:**
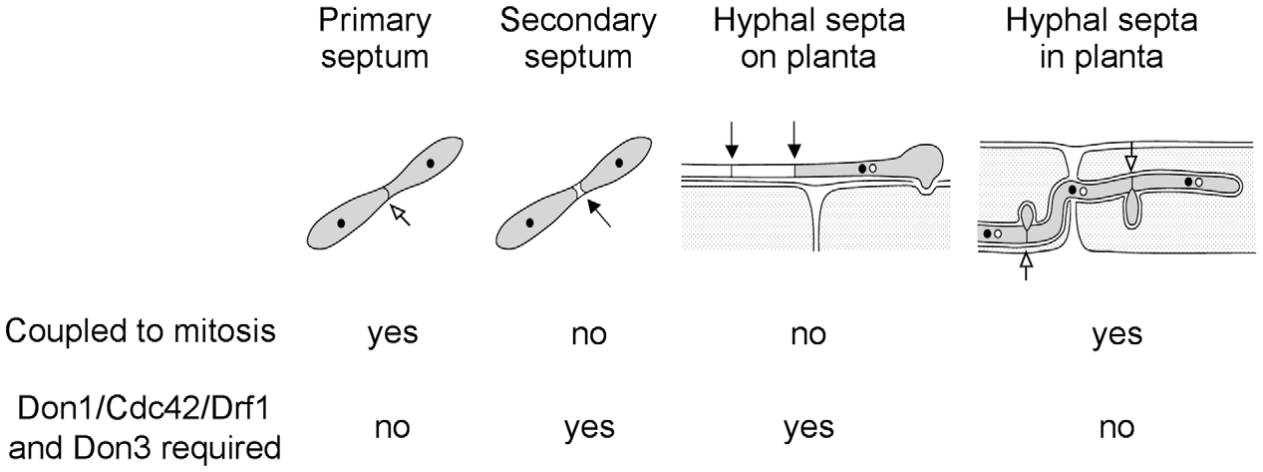
The Cdc42 signaling network regulates septation events that are uncoupled to mitosis. During the life cycle of *U. maydis* the coordination of septum formation can be either coupled to or uncoupled from mitosis. If septation is independent of mitosis it requires the signaling network consisting of Don1/Cdc42/Drf1 and Don3.

In this study we investigated four different events of septum formation in the life cycle of *U. maydis*. During budding growth formation of the primary septum is coupled to mitosis, while the secondary septum permits cell separation (Figure 10) [22]. We have shown previously that secondary septum formation requires Cdc42, Don1 and Don3 [19], [22]. G2 arrested infectious hyphae form septa, which are not coupled to mitosis. Here we could demonstrate that the Don1/Cdc42/Drf1/Don3 signaling network is not only required for formation of the secondary septum in budding cells, but also during septation of infectious hyphae (Figure 10). Finally, we investigated septum formation of proliferating hyphae inside the plant, a septation event that is coupled to mitosis [41]. We found that the Cdc42 signaling network is not required during this developmental stage and the ability to form septa returns immediately after plant penetration when cell-cycle arrest is released. Consequently, *U. maydis* uses at least two different mechanisms to produce septa, a mitosis uncoupled mechanism, which requires Cdc42 and a mitosis coupled mechanism, which does not require Cdc42 (Figure 10). The specific importance of the described Cdc42 signaling module during non-mitotic septation is shown by the distinct phenotypes of *drf1* mutants. Drf1 is only needed for the formation of the actomyosin ring during formation of the secondary septum and during hyphal septation albeit the contractile actomyosin ring is already formed during primary septation coupled to mitosis of haploid budding cells. It is well established that the GTPase RhoA defines the division site of eukaryotic cells and requires spindle microtubules [62]. How the Don1/Cdc42/Drf1 module coordinates the positioning of hyphal septa in G2 arrested filaments and secondary septa during budding growth is yet unclear but remains an exciting topic to be elucidated. Especially how the regularity of septa in filaments is established would be interesting to understand [11]. Is the position of the nucleus important or could changes in cytoplasmic volume account for this regularity?

Taken together, our data provide new insights into the role of septation during pathogenic development of *U. maydis*. Cell cycle independent septation is unusual among eukaroytes [63], which makes infectious hyphae of *U. maydis* a valuable model system to study this kind of septation.

## Methods

### Strains

*Escherichia coli* strain DH5a and Top10 were used for cloning and amplification of plasmid DNA. *U. maydis* strains FB1, Bub8, AB31 [32] and SG200AM1 [12] were utilized as wild type backgrounds for all strains created for this manuscript. FB1Δ*don3*, Bub8Δ*don3*, FB1Δ*don1*, Bub8Δ*don1*, FB1Δ*cdc42* and Bub8Δ*cdc42* have been described elsewhere [19], [22], [35]. All strains used are listed in table S1.

### Alignment

According to the NCBI database these reference sequences were used: *M. musculus*: MmDrf1: NP_031884; *U. maydis*: Srf1 (um12254): XP_760288, UmDrf1 (um01141): XP_757288.

### Generation of strains

In general, transformation of *U. maydis* was performed as described [64]. For expression studies constructs expressing *drf1* or *drf1*Δ*GBD* under control of the *etef*-promoter were integrated into the *cbx-*locus by homologous recombination [65]. Deletion mutants and the endogenous *gfp*-fusion of *cdc15* were generated as described [66]. For septin localization in the AB31 and the AB31Δ*drf1* strain the Cdc10-RFP construct was used as described previously [38].

### Plasmid construction

For overexpression in *U. maydis* the open reading frame of *drf1* (6588 bp) was cloned into the SmaI- and NotI-sites of pETEF-MXN-GFP, which is derived from p123 and resulted in pETEF-drf1 [67]. The GBD (aa 534–886) was deleted from this plasmid to generate pETEF-drf1ΔGBD. For the C-terminal fusion of GFP the ORF of Drf1ΔGBD was cloned in the NcoI-site of pETEF-MXN-GFP to generate pETEF-drf1ΔGBD-GFP. For exocyst localization in *U. maydis* the ORF of Sec4 (um03865) was amplified and cloned into p123 to generate p123-GFP-Sec4. All constructs have been confirmed by sequencing. Primer sequences will be supplied by the corresponding author (B. S.) on request. Deletion constructs for *drf1* and *don1* were generated using the SfiI-technique as described previously [66].

### GST pull-down experiment

An excess of bacterial expressed GST or GST-Cdc42 proteins was immobilized on glutathione resin according the manufacturer's instructions (Macherey-Nagel) and pretreated with either 1 mM GDP or 1 mM GTPgS in the presence of 2 mM EDTA for 1 h at 4°C. The GTP/GDP loading was stopped with 40 mM MgCl_2_ and washed three-times with TBS. *U. maydis* protein extract was made as described [68] and directly used for the pull-down for 16 h at 4°C. GFP-fusion proteins were detected using a GFP-monoclonal antibody from Santa Cruz Biotechnology (sc-9996).

### Analysis of appressoria

The in vitro system for inducing filaments and appressoria in *U. maydis* was applied as described previously [12] with minor modifications. Briefly, SG200AM1 and derivative strains were grown in YEPSL medium (0.4% yeast extract, 0.4% peptone, 2% sucrose) at 28°C to an OD_600_ of 0.8. The cells were resuspended in 2% YEPSL to an OD_600_ of 0.2 and supplemented with 100 µM (f.c.) 16-hydroxyhexadecanoic acid (Sigma-Aldrich). Cells were sprayed (EcoSpray Labo Chimie, France) on Parafilm M and incubated by 100% humidity at 28°C for 20 h. The samples were stained by calcofluor white to visualize fungal cells and the ratio of filamentous cells expressing the AM1 marker (indicative for appressorium formation) to the total amount of filaments was determined using fluorescence microscopy. The experiment was done in three biological replicates.

The diameter of appressoria was measured using the ImageJ software (NIH, USA).

For examination of appressoria on the leaf surface, AM1 derivatives were inoculated into seven-day-old maize seedlings 1 cm above ground. After 20 hours the third oldest leaf was prepared, washed in water and incubated for 30 s with calcofluor white. Appressorium formation was quantified as described above.

### Plant infections

Solopathogenic strains or compatible haploid strains were grown in YEPSL to an OD_600_ of 0.8 and concentrated in H_2_O to a final OD_600_ of 1.0. This suspension was used to infect seven-day-old seedlings of Early Golden Bantam (Olds Seeds, Madison) by injecting 0.5 ml into each seedling. 12 days after infection disease symptoms were evaluated according to the disease rating criteria reported by Kämper et al. [9]. The experiment was done in three biological replicates.

### Plate and drop mating assays

Plate-mating assays were performed by placing mixtures of compatible strains on PD-plates containing 1% activated charcoal followed by incubation at room temperature [33]. For filament formation analysis a drop of such mixture was covered by a small glass platelet allowing filamentous growth onto the glass surface. After two and three days the platelets were analyzed on a microscope slide.

### Microscopy

*U. maydis* cells from logarithmically growing cultures were placed on agarose cushions. Cells were visualized by differential interference contrast (DIC) and epifluorescence microscopy using a Zeiss Axiophot 200 microscope (Göttingen, Germany). Calcofluor white staining was performed as described previously [22]. Briefly, to stain fungal material, samples were incubated in Calcofluor Fluorescent Brightner 28 (100 µg/ml in 0.2 M Tris/HCl, pH 8; Sigma-Aldrich) for 30 s. Images were taken using a cooled CCD camera (Hamamatsu Orca-ER, Herrsching, Germany) with an exposure time of 50–300 ms. Image acquisition and deconvolution were performed using Improvision Volocity software (Perkin-Elmer, Rodgau, Germany) and processing was carried out with Photoshop (Adobe) and ImageJ (NIH, USA).

The intensity of calcofluor white staineing of secondary septa was analyzed using ImageJ (NIH, USA).

To investigate septum formation of fungal hyphae inside the plant, three days after infection the third oldest leaf was destained in ethanol, transferred to 10% KOH, incubated at 85°C for 4 hours, washed three times with PBS buffer (140 mM NaCl, 16 mM Na_2_HPO_4_, 2 mM KH_2_PO_4_, 3.5 mM KCl, and 1 mM Na_2_-EDTA, pH 7.4), and incubated under vacuum in staining solution (10 µg/ml propidium iodide and 10 µg/ml WGA-AF 488 in PBS, pH 7.4) according to Doehlemann et al. [57]. WGA-AF 488 was purchased from Invitrogen, propidium iodide from Sigma-Aldrich. Images were taken and processed as described above.

Confocal microscopy was performed using a TCS-SP5 confocal microscope (Leica Microsystems). For GFP fluorescence, an excitation of 488 nm and detection at 495–530 nm was used. Propidium iodide fluorescence was excited with 561 nm and detected at 580–630 nm. To visualize WGA-AF 488, an excitation of 488 nm and detection at 500–540 nm was used. Calcofluor white was excited with a 405 nm laser and detected at 415–460 nm. Images were processed using LAS-AF software (Leica Microsystems).

### Nucleic acid procedures and quantitative real time PCR

For RNA isolation the *U. maydis* strain AB31 was grown 7 hours in 2% glucose or arabinose containing YNB-liquid media. 25 ml culture was harvested and ground in liquid nitrogen. RNA was extracted with Trizol (Invitrogen, Karlsruhe, Germany) and purified using RNeasy kit (Qiagen, Hilden, Germany). For qRT-PCR cDNA was synthesised using the First Strand cDNA kit (Fermentas, Mannheim, Germany) employing 1 µg total RNA. qRT-PCR was performed using the MAXIMA SYBR-Green qPCR Master-Mix (Fermentas, Mannheim, Germany) on a Biorad iCycler. Reaction conditions were as follows: 5 min 95°C followed by 45 cycles of 15 sec 95°C/15 sec 60°C/30 sec 72°C. Primers used for amplification were for the control gene: TCGACATCGTCAAGGCTATC (5′*ppi* RT) and CGATGGTGATCTTGGACTTG (3′*ppi* RT). To amplify a *drf1*-fragment we used: TCAGGGTCAAGCAAAGTCAG (5′*drf1* RT) and GTCAGTCATTCGAGATCGT (3′*drf1* RT). Ratios of *drf1*/*ppi* were calculated for each timepoint and the glucose value was set to 1.

### Statistical analysis

Data are expressed as means ±SD of triplicate samples. Statistical significance was assessed using Statistical Calculators (www.danielsoper.com) and considered significant if p values were <0.05. We performed a Wilcoxon rank-sum test to compare the distributions of disease symptoms induced by *U. maydis* strains. For this purpose we used a web-calculator (http://faculty.vassar.edu/lowry/wilcoxon.html).

### Accession numbers

UM accession numbers are from MUMDB (http://mips.helmholtz-muenchen.de/genre/proj/ustilago/) and XP/XM/NP accession numbers are from NCBI (http://blast.ncbi.nlm.nih.gov/Blast.cgi): Drf1 (um01141) XP_757288, Don1 (um10152) XP_758565, Don3 (um05543) XP_761690, Cdc42 (um00295) XP_756442, Cdc15 (um00168) XP_756315, Cdc10 (um10644) XP_759364, Sec4 (um03865) XP_760012, Ppi (um03726) XM_754780, Srf1 (um12254) XP_760288, mmDrf1 NP_031884.

## Supporting Information

Figure S1 Retraction septa formation is abolished in *drf1* mutants. The diagram represents the average number of retraction septa in different wt and *drf1* mutant strains after the indicated time points. The number of investigated filaments is indicated. Errorbars indicate standard deviation. #: The on planta number of retraction septa is difficult to count due to the texture of the leaf surface, but is at least 10 per filament after 20 hours.
(TIFF)

Figure S2 Expression of GFP-Sec4 in wild type and Δ*drf1* filaments. The figure shows the comparison of GFP-Sec4 distribution in wild type (upper panel) and Δ*drf1* filaments (lower panels). Sec4 localizes at vesicles distributed all over the filaments. The density of vesicles is reduced in longer filaments of *drf1* mutants.
(TIFF)

Figure S3 Don1 and Don3 are required for hyphal septation. For filament analysis haploid *don1* or *don3* mutant cells were mixed with the compatible mating partner bearing the same deletion. Filaments were stained with calcofluor white (CF). To demonstrate the length of the filaments it was necessary to combine two to three images. (Scale bars: 10 µm)
(TIFF)

Figure S4 Complementation of *drf1* mutant cells by Drf1 and Drf1ΔGBD. A-C: In *drf1* mutants overexpressing Drf1 (A) and Drf1ΔGBD (B,C) cell separation (DIC) and deposition of cell wall material (CF) as well retraction septa formation were completely restored. D: The GFP fusion to Drf1ΔGBD was detected in dots distributed allover the cell and during contraction of the secondary CAR (green). (Scale bars: 10 µm)
(TIFF)

Figure S5 Constitutive expression of Drf1 is not able to suppress the *cdc42* deletion strain. Δ*cdc42*+pETEF-Drf1ΔGBD was grown to an OD_600_ = 0.5 and was stained with calcofluor white (left image). For the colony morphology image single cells were grown for three days on YEPS with 1.3% Agar.
(TIFF)

Figure S6 CAR formation is impaired in hyphae of *don1* or *don3* mutants. AB31Δ*don1* and AB31Δ*don3* Petef:don3^M157A^ expressing endogenously Cdc15-GFP (green) were grown on arabinose to induce hyphal growth for 6 h. For AB31Δ*don3* Petef:don3^M157A^ kinase activity was blocked using 1 µM NA-PP1. Cells were co-stained with calcofluor white (CF, red). The region, where normally the first distal septum is expexcted is magnified.
(TIFF)

Figure S7 CAR formation is not observed at the site of secondary septum in *drf* mutants. CAR formation in haploid cells is shown for wt and for Δ*drf1* cells using Cdc15-GFP (green). Cells were co-stained with calcofluor white (CF, red). Stars indicate the primary septum. Arrows indicate the site of secondary septum formation.
(TIFF)

Figure S8 Septin rings depend on Drf1 during retraction septa formation. A: Cdc10-RFP is part of the septin ring during retraction septum formation in AB31 filaments independent of length. B: In short AB31Δ*drf1* filaments septin collars are formed at the bud neck. C: Long AB31Δ*drf1* filaments lack any specific septin structure. The length of each filament is measured using ImageJ and indicated at the bottom of the DIC image. (Scale bars: 10 µm)
(TIFF)

Figure S9 *drf1, don1 and don3* mutants show reduced appressoria formation on the leaf surface. Seven-day-old maize seedlings were infected with SG200AM1 and the respective derivative strains. 20 h after infection the surface of the third leaf was analyzed by confocal microscopy. Fungal material was stained with calcofluor white (blue) and expression of the AM1 gfp-reporter (green) indicates appressorium formation (see arrows). The overlays of maximum projections of both channels with the corresponding bright-field image are depicted.
(TIFF)

Figure S10 Filament length distribution is similar in SG200 AM1 and the *drf1* mutant. The same images analyzed for figure 6 were used to measure the length of all filaments.
(TIFF)

Figure S11 Appressoria diameter is similar in SG200 AM1 and the *drf1* mutant. The same images analyzed for figure 6 were used to measure the diameter of the appressoria. Errorbars indicate standard deviation.
(TIFF)

Table S1 *U. maydis* strains used in this study.
(DOC)

Protocol S1 Inhibition of the analog-sensitive Don3 kinase.
(DOC)

## References

[1] Emmett RW, Parbery DG (1975). Appressoria..

[2] Tucker SL, Talbot NJ (2001). Surface attachment and pre-penetration stage development by plant pathogenic fungi.. Annu Rev Phytopathol.

[3] Bechinger C, Giebel KF, Schnell M, Leiderer P, Deising HB (1999). Optical measurements of invasive forces exerted by appressoria of a plant pathogenic fungus.. Science.

[4] Dixon KP, Xu JR, Smirnoff N, Talbot NJ (1999). Independent signaling pathways regulate cellular turgor during hyperosmotic stress and appressorium-mediated plant infection by *Magnaporthe grisea*.. Plant Cell.

[5] Kumamoto CA (2008). Molecular mechanisms of mechanosensing and their roles in fungal contact sensing.. Nat Rev Microbiol.

[6] Wilson RA, Talbot NJ (2009). Under pressure: investigating the biology of plant infection by *Magnaporthe oryzae*.. Nat Rev Microbiol.

[7] Lanver D, Mendoza-Mendoza A, Brachmann A, Kahmann R (2010). Sho1 and Msb2-related proteins regulate appressorium development in the smut fungus *Ustilago maydis*.. Plant Cell.

[8] Banuett F (1992). *Ustilago maydis*, the delightful blight.. Trends Genet.

[9] Kämper J, Kahmann R, Bölker M, Ma LJ, Brefort T (2006). Insights from the genome of the biotrophic fungal plant pathogen *Ustilago maydis*.. Nature.

[10] Garcia-Muse T, Steinberg G, Pérez-Martin J (2003). Pheromone-induced G2 arrest in the phytopathogenic fungus *Ustilago maydis*.. Eukaryot Cell.

[11] Steinberg G, Schliwa M, Lehmler C, Bölker M, Kahmann, R (1998). Kinesin from the plant pathogenic fungus *Ustilago maydis* is involved in vacuole formation and cytoplasmic migration.. J Cell Sci.

[12] Mendoza-Mendoza A, Berndt P, Djamei A, Weise C, Linne U (2009). Physical-chemical plant-derived signals induce differentiation in *Ustilago maydis*.. Mol Microbiol.

[13] Schirawski J, Böhnert HU, Steinberg G, Snetselaar K, Adamikowa L (2005). Endoplasmic reticulum glucosidase II is required for pathogenicity of *Ustilago maydis*.. Plant Cell.

[14] Banuett F, Herskowitz I (1994). Morphological transitions in the life cycle of *Ustilago maydis* and their genetic control by the *a* and *b* loci.. Exp Mycol.

[15] Bölker M, Urban M, Kahmann R (1992). The a mating type locus of *U. maydis* specifies cell signaling components.. Cell.

[16] Kahmann R, Bölker M (1996). Self/nonself recognition in fungi: old mysteries and simple solutions.. Cell.

[17] Kämper J, Reichmann M, Romeis T, Bölker M, Kahmann R (1995). Multiallelic recognition: nonself-dependent dimerization of the bE and bW homeodomain proteins in *Ustilago maydis*.. Cell.

[18] Heimel K, Scherer M, Schuler D, Kämper J (2010). The *Ustilago maydis* Clp1 protein orchestrates pheromone and b-dependent signaling pathways to coordinate the cell cycle and pathogenic development.. Plant Cell.

[19] Mahlert M, Leveleki L, Hlubek A, Sandrock B, Bölker M (2006). Rac1 and Cdc42 regulate hyphal growth and cytokinesis in the dimorphic fungus *Ustilago maydis*.. Mol Microbiol.

[20] Hlubek A, Schink KO, Mahlert M, Sandrock B, Bölker M (2008). Selective activation by the guanine nucleotide exchange factor Don1 is a main determinant of Cdc42 signaling specificity in *Ustilago maydis*.. Mol Microbiol.

[21] Sandrock B, Böhmer C, Bölker M (2006). Dual function of the germinal centre kinase Don3 during mitosis and cytokinesis in *Ustilago maydis*.. Mol Microbiol.

[22] Weinzierl G, Leveleki L, Hassel A, Kost G, Wanner G (2002). Regulation of cell separation in the dimorphic fungus *Ustilago maydis*.. Mol Microbiol.

[23] Higgs HN (2005). Formin proteins: a domain-based approach.. Trends Biochem Sci.

[24] Kovar DR, Harris ES, Mahaffy R, Higgs HN, Pollard TD (2006). Control of the assembly of ATP- and ADP-actin by formins and profilin.. Cell.

[25] Pruyne D, Evangelista M, Yang C, Bi E, Zigmond S (2002). Role of formins in actin assembly: nucleation and barbed-end association.. Science.

[26] Liu W, Sato A, Khadka D, Bharti R, Diaz H (2008). Mechanism of activation of the Formin protein Daam1.. Proc Natl Acad Sci U S A.

[27] Sagot I, Rodal AA, Moseley J, Goode BL, Pellman D (2002). An actin nucleation mechanism mediated by Bni1 and profilin.. Nat Cell Biol.

[28] Castrillon DH, Wasserman SA (1994). Diaphanous is required for cytokinesis in Drosophila and shares domains of similarity with the products of the limb deformity gene.. Development.

[29] Chalkia D, Nikolaidis N, Makalowski W, Klein J, Nei M (2008). Origins and evolution of the formin multigene family that is involved in the formation of actin filaments.. Mol Biol Evol.

[30] Evangelista M, Zigmond S, Boone C (2003). Formins: signaling effectors for assembly and polarization of actin filaments.. J Cell Sci.

[31] Li F, Higgs HN (2003). The mouse Formin mDia1 is a potent actin nucleation factor regulated by autoinhibition.. Curr Biol.

[32] Brachmann A, Weinzierl G, Kämper J, Kahmann R (2001). Identification of genes in the bW/bE regulatory cascade in *Ustilago maydis*.. Mol Microbiol.

[33] Day PR, Anagnostakis SL (1971). Corn smut dikaryon in culture.. Nat New Biol.

[34] Snetselaar KM, Mims CW (1992). Sporidial fusion and infection of maize seedlings by the smut fungus *Ustilago maydis*.. Mycologia.

[35] Schmitz HP, Kaufmann A, Köhli M, Laissue PP, Philippsen P (2006). From function to shape: a novel role of a formin in morphogenesis of the fungus *Ashbya gossypii*.. Mol Biol Cell.

[36] Böhmer C, Böhmer M, Bölker M, Sandrock B (2008). Cdc42 and the Ste20-like kinase Don3 act independently in triggering cytokinesis in *Ustilago maydis*.. J Cell Sci.

[37] Evangelista M, Blundell K, Longtine MS, Chow CJ, Adames N (1997). Bni1p, a yeast formin linking cdc42p and the actin cytoskeleton during polarized morphogenesis.. Science.

[38] Böhmer C, Ripp C, Bölker M (2009). The germinal centre kinase Don3 triggers the dynamic rearrangement of higher-order septin structures during cytokinesis in *Ustilago maydis*.. Mol Microbiol.

[39] Alvarez-Tabares I, Perez-Martin J (2010). Septins from the phyotpathogenic fungus *Ustilago maydis* are required for proper morphogenesis but dispensable for virulence.. PLoS One.

[40] Spellig T, Bottin A, Kahmann R (1996). Green fluorescent protein (GFP) as a new vital marker in the phytopathogenic fungus *Ustilago maydis*.. Mol Gen Genet.

[41] Scherer M, Heimel K, Starke V, Kämper J (2006). The Clp1 protein is required for clamp formation and pathogenic development of *Ustilago maydis.*. Plant Cell.

[42] Evangelista M, Pruyne D, Amberg DC, Boone C, Bretscher A (2002). Formins direct Arp2/3-independent actin filament assembly to polarize cell growth in yeast.. Nat Cell Biol.

[43] Kikyo M, Tanaka K, Kamei T, Ozaki K, Fujiwara T (1999). An FH domain-containing Bnr1p is a multifunctional protein interacting with a variety of cytoskeletal proteins in *Saccharomyces cerevisiae.*. Oncogene.

[44] Pruyne D, Bretscher A (2000). Polarization of cell growth in yeast. The role of the cortical actin cytoskeleton.. J Cell Sci.

[45] Imamura H, Tanaka K, Hihara T, Umikawa M, Kamei T (1997). Bni1p and Bnr1p: downstream targets of the Rho family small G-proteins which interact with profilin and regulate actin cytoskeleton in *Saccharomyces cerevisiae*.. EMBO J.

[46] Kamei T, Tanaka K, Hihara T, Umikawa M, Imamura H (1998). Interaction of Bnr1p with a novel Src homology 3 domain-containing Hof1p. Implication in cytokinesis in *Saccharomyces cerevisiae*.. J Biol Chem.

[47] Bathe M, Chang F (2010). Cytokinesis and the contractile ring in fission yeast: towards a systems-level understanding.. Trends Microbiol.

[48] Chang F, Woollard A, Nurse P (1996). Isolation and characterization of fission yeast mutants defective in the assembly and placement of the contractile actin ring.. J Cell Sci.

[49] Chang F, Drubin D, Nurse P (1997). cdc12p, a protein required for cytokinesis in fission yeast, is a component of the cell division ring and interacts with profiling.. J Cell Biol.

[50] Yonetani A, Chang F (2010). Regulation of Cytokinesis by the forming Cdc12p.. Curr Biol.

[51] Olson MF (2003). Dispatch. GTPase signaling: new functions for Diaphanous-related formins.. Curr Biol.

[52] Schirenbeck A, Bretschneider T, Arasada R, Schleicher M, Faix J (2005). The Diaphanous-related formin dDia2 is required for the formation and maintenance of filopodia.. Nat Cell Biol.

[53] Robinow CF (1963). Observations on cell growth, mitosis, and division in the fungus *Basidiobolus ranarum*.. J Cell Biol.

[54] Ingold CT (1982). Retraction-septa in various fungi.. Trans Br Mycol Soc.

[55] de Jong JC, McCormack BJ, Smirnoff N, Talbot NJ (1997). Glycerol generates turgor in rice blast.. Nature.

[56] Choi W, Dean RA (1997). The adenylate cyclase gene MAC1 of *Magnaporthe grisea* controls appressorium formation and other aspects of growth and development.. Plant Cell.

[57] Döhlemann G, Wahl R, Vranes M, de Vries RP, Kämper J (2008). Establishment of compatibility in the *Ustilago maydis*/maize pathosystem.. J Plant Physiol 165,.

[58] Veneault-Fourrey C, Barooah M, Egan M, Wakley G, Talbot NJ (2006). Autophagic fungal cell death is necessary for infection by the rice blast fungus.. Science.

[59] Kershaw MJ, Talbot NJ (2009). Genome-wide functional analysis reveals that infection-assciated fungal autophagy is essential for rice blast disease.. Proc Natl Acad Sci U S A.

[60] Saunders DG, Dagdas YF, Talbot NJ (2010). Spatial uncoupling of mitosis and cytokinesis during appressorium-mediated plant infection by the rice blast fungus *Magnaporthe oryzae.*. Plant Cell.

[61] Christensen JJ (1963). Corn smut caused by *Ustilago maydis*..

[62] Wadsworth P (2005). Cytokinesis: rho marks the spot.. Curr Biol.

[63] Oliferenko S, Chew TG, Balasubramanian MK (2009). Positioning cytokinesis.. Genes Dev.

[64] Schulz B, Banuett F, Dahl M, Schlesinger R, Schäfer W (1990). The *b* alleles of *U. maydis*, whose combinations program pathogenic development, code for polypeptides containing a homeodomain-related motif.. Cell.

[65] Loubradou G, Brachmann A, Feldbrügge M, Kahmann R (2001). A homologue of the transcriptional repressor Ssn6p antagonizes cAMP signaling in *Ustilago maydis*.. Mol Microbiol.

[66] Brachmann A, König J, Julius C, Feldbrügge M (2004). A reverse genetic approach for generating gene replacement mutants in *Ustilago maydis*.. Mol Genet Genomics.

[67] Bottin A, Kämper J, Kahmann R (1996). Isolation of a carbon source-regulated gene from *Ustilago maydis*.. Mol Gen Genet.

[68] Böhmer M, Colby T, Böhmer C, Bräutigam A, Schmidt J (2007). Proteomic analysis of dimorphic transition in the phytopathogenic fungus *Ustilago maydis*.. Proteomics.

