# Supplementary figures and images for: Septation of Infectious Hyphae Is Critical for Appressoria Formation and Virulence in the Smut Fungus Ustilago Maydis
Source: PLoS Pathog. 2011 May 19;7(5):e1002044. doi: 10.1371/journal.ppat.1002044 (PMC3098242; doi:10.1371/journal.ppat.1002044)

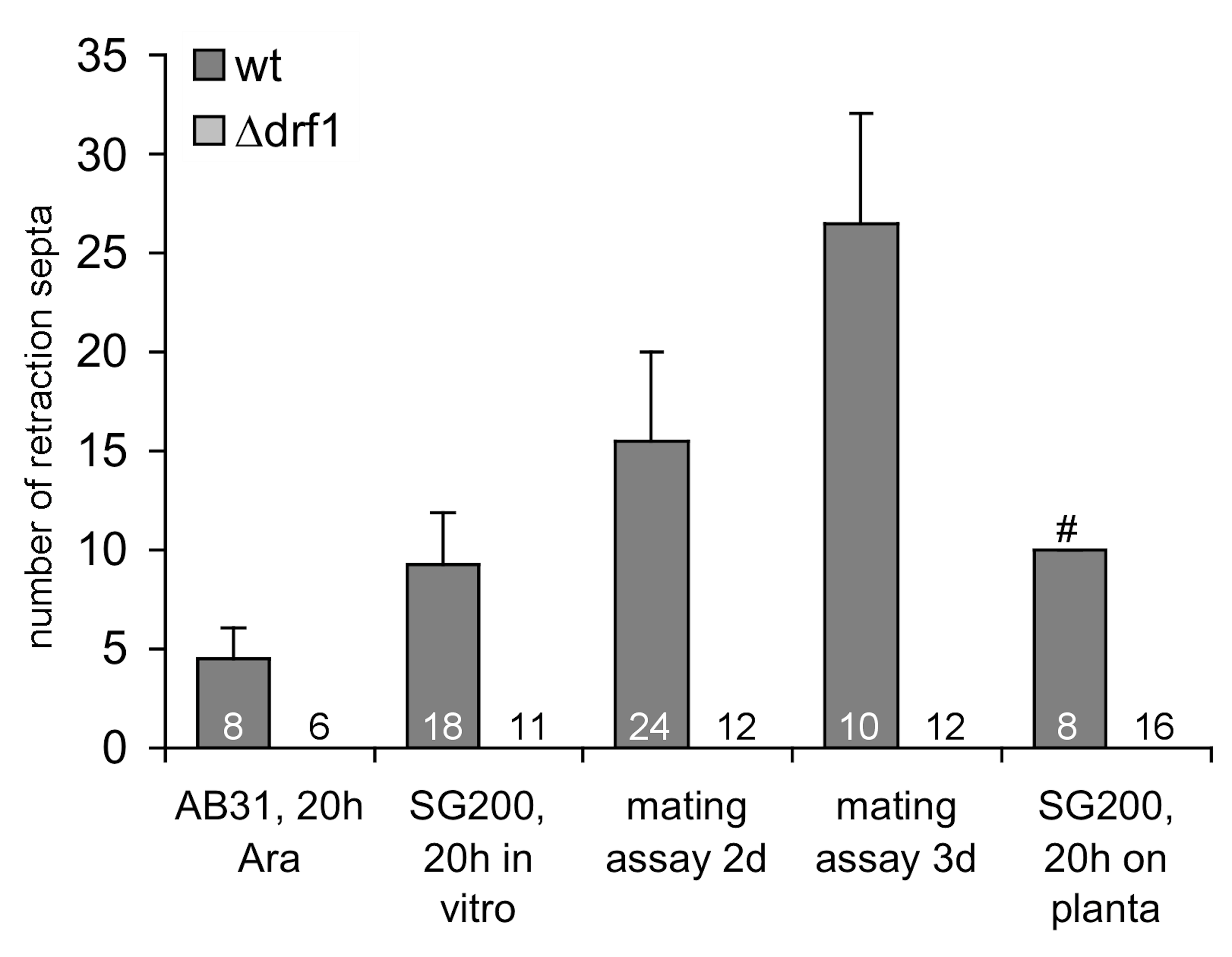

Supplement: Figure S1 — Retraction septa formation is abolished in drf1 mutants. The diagram represents the average number of retraction septa in different wt and drf1 mutant strains after the indicated time points. The number of investigated filaments is indicated. Errorbars indicate standard deviation. #: The on planta number of retraction septa is difficult to count due to the texture of the leaf surface, but is at least 10 per filament after 20 hours. (TIFF) [file ppat.1002044.s001.tif]

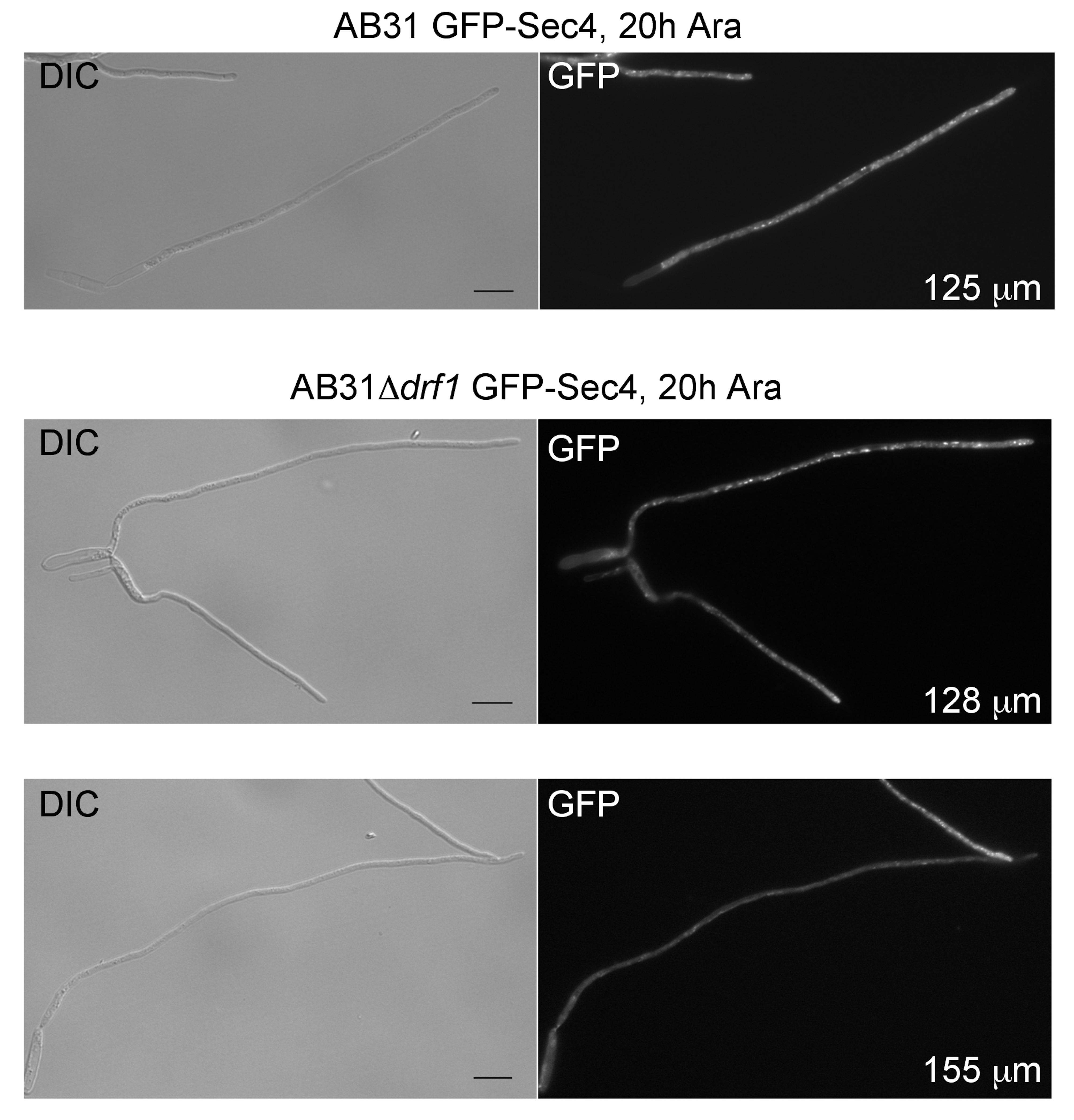

Supplement: Figure S2 — Expression of GFP-Sec4 in wild type and Δdrf1 filaments. The figure shows the comparison of GFP-Sec4 distribution in wild type (upper panel) and Δdrf1 filaments (lower panels). Sec4 localizes at vesicles distributed all over the filaments. The density of vesicles is reduced in longer filaments of drf1 mutants. (TIFF) [file ppat.1002044.s002.tif]

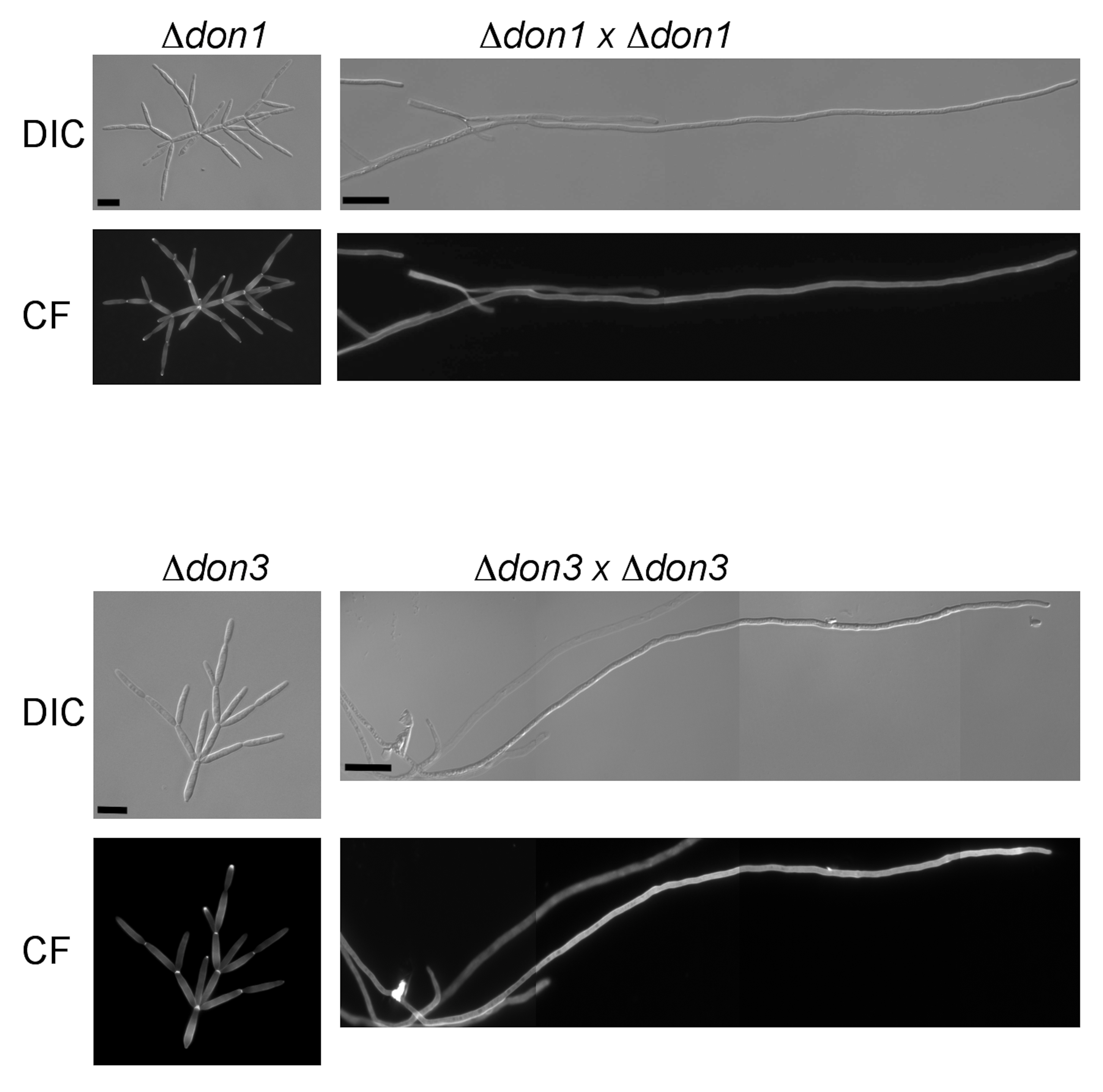

Supplement: Figure S3 — Don1 and Don3 are required for hyphal septation. For filament analysis haploid don1 or don3 mutant cells were mixed with the compatible mating partner bearing the same deletion. Filaments were stained with calcofluor white (CF). To demonstrate the length of the filaments it was necessary to combine two to three images. (Scale bars: 10 µm) (TIFF) [file ppat.1002044.s003.tif]

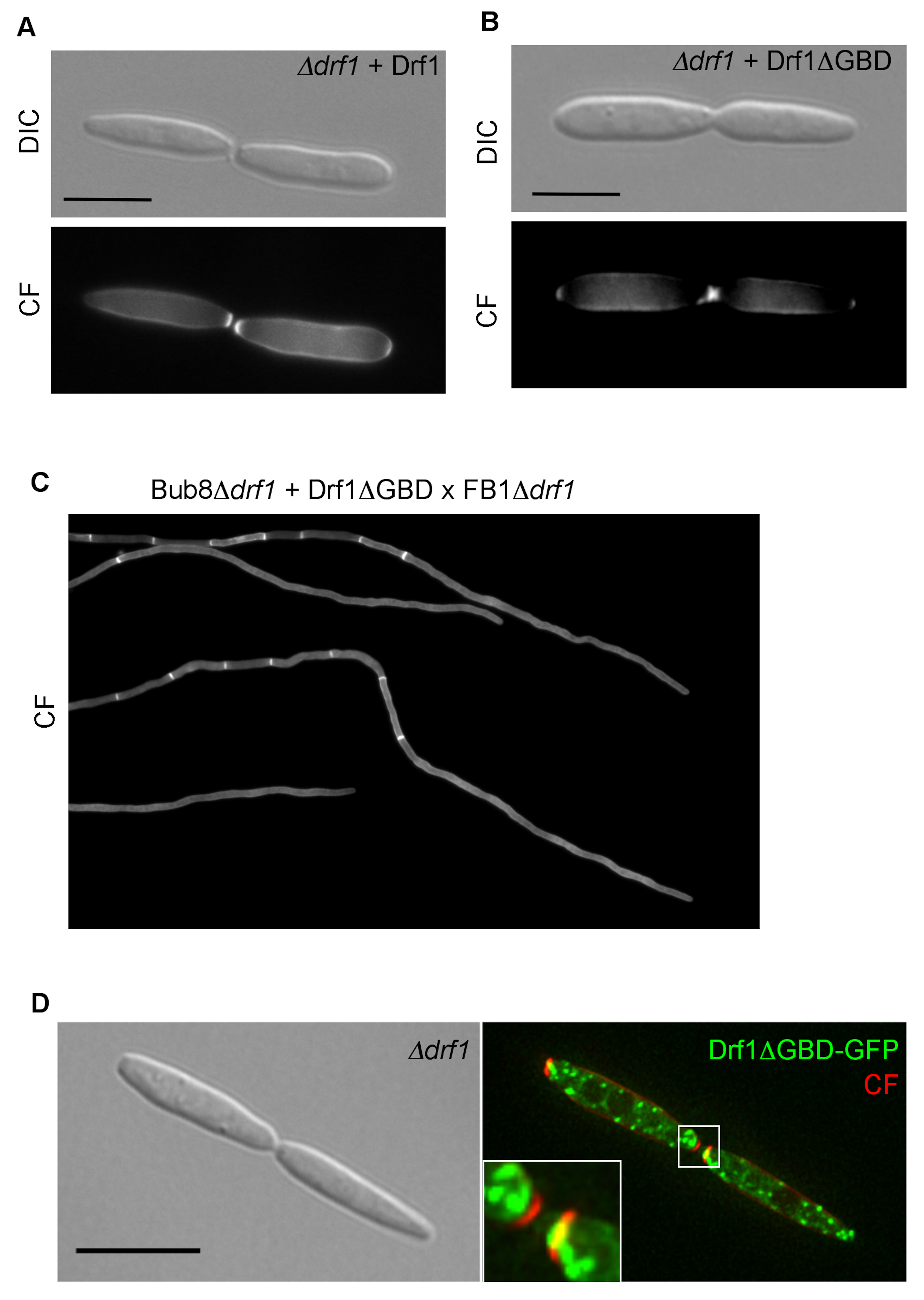

Supplement: Figure S4 — Complementation of drf1 mutant cells by Drf1 and Drf1ΔGBD. A-C: In drf1 mutants overexpressing Drf1 (A) and Drf1ΔGBD (B,C) cell separation (DIC) and deposition of cell wall material (CF) as well retraction septa formation were completely restored. D: The GFP fusion to Drf1ΔGBD was detected in dots distributed allover the cell and during contraction of the secondary CAR (green). (Scale bars: 10 µm) (TIFF) [file ppat.1002044.s004.tif]

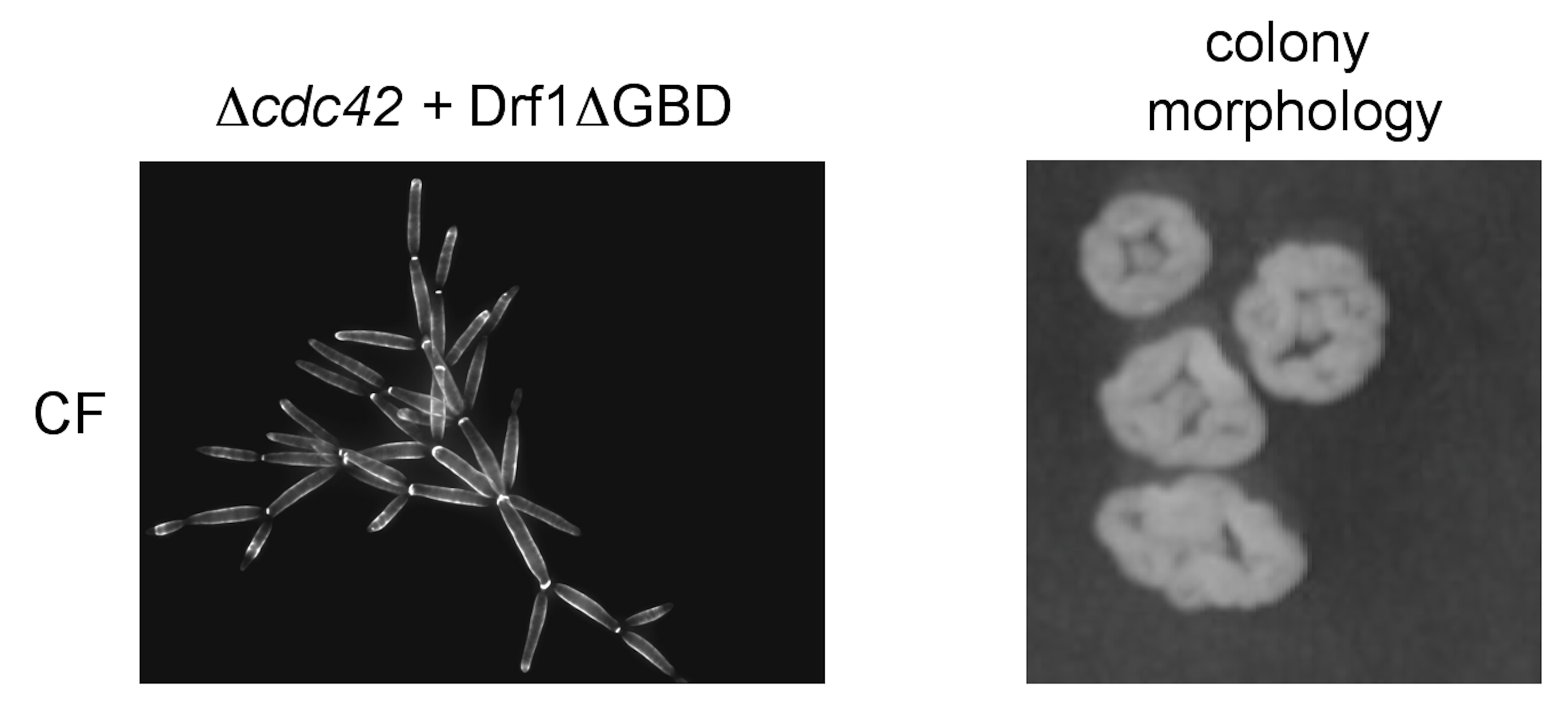

Supplement: Figure S5 — Constitutive expression of Drf1 is not able to suppress the cdc42 deletion strain. Δcdc42+pETEF-Drf1ΔGBD was grown to an OD600 = 0.5 and was stained with calcofluor white (left image). For the colony morphology image single cells were grown for three days on YEPS with 1.3% Agar. (TIFF) [file ppat.1002044.s005.tif]

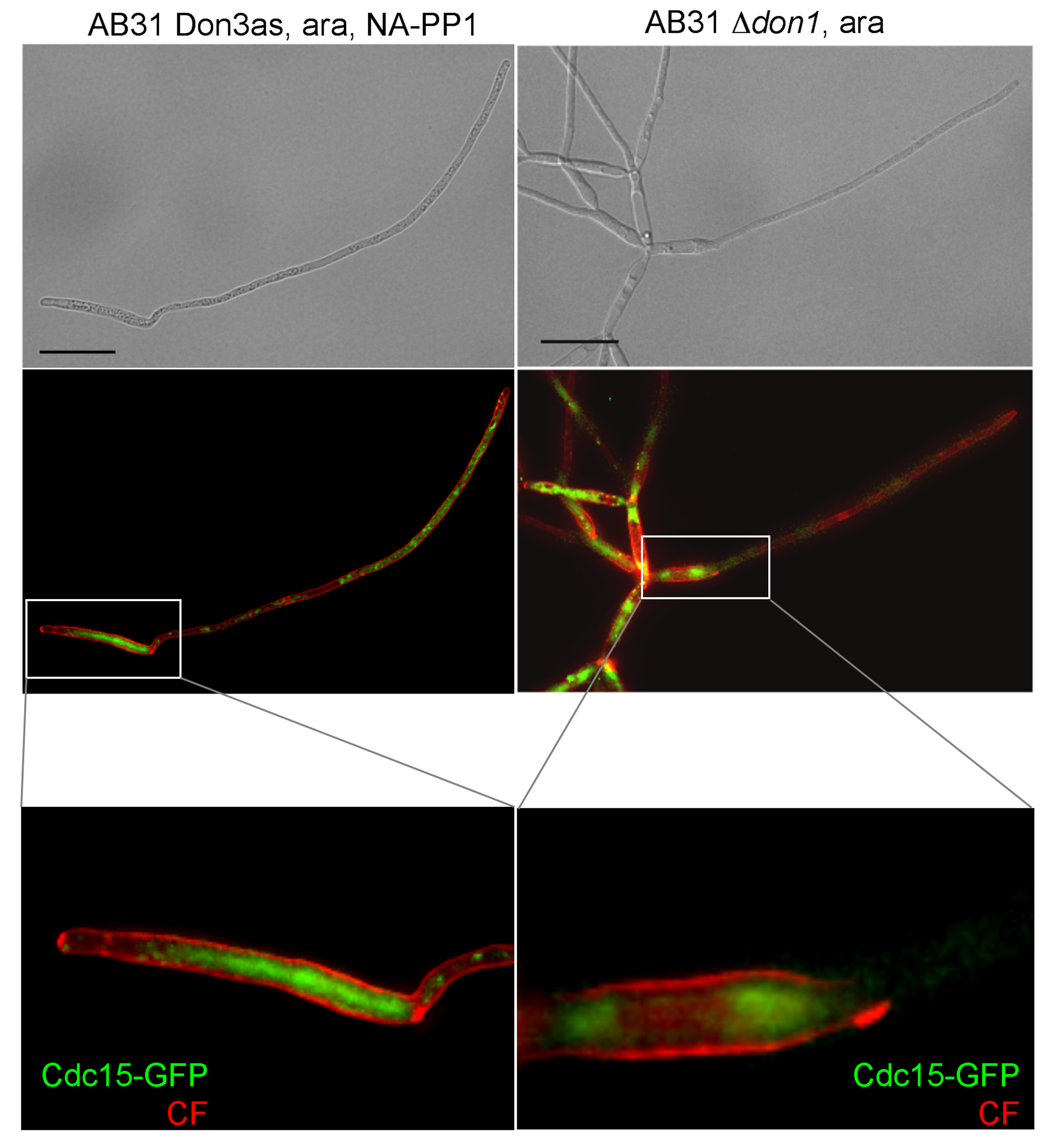

Supplement: Figure S6 — CAR formation is impaired in hyphae of don1 or don3 mutants. AB31Δdon1 and AB31Δdon3 Petef:don3M157A expressing endogenously Cdc15-GFP (green) were grown on arabinose to induce hyphal growth for 6 h. For AB31Δdon3 Petef:don3M157A kinase activity was blocked using 1 µM NA-PP1. Cells were co-stained with calcofluor white (CF, red). The region, where normally the first distal septum is expexcted is magnified. (TIFF) [file ppat.1002044.s006.tif]

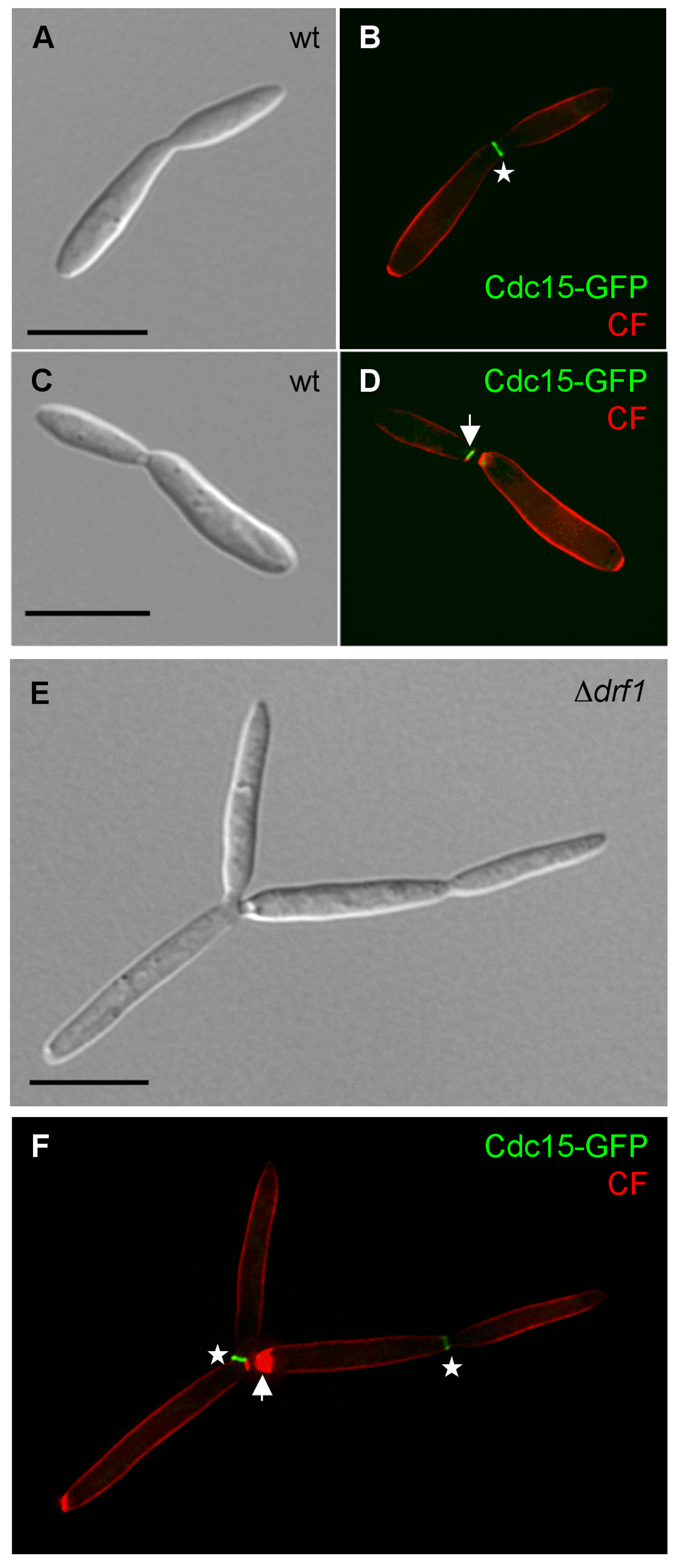

Supplement: Figure S7 — CAR formation is not observed at the site of secondary septum in drf mutants. CAR formation in haploid cells is shown for wt and for Δdrf1 cells using Cdc15-GFP (green). Cells were co-stained with calcofluor white (CF, red). Stars indicate the primary septum. Arrows indicate the site of secondary septum formation. (TIFF) [file ppat.1002044.s007.tif]

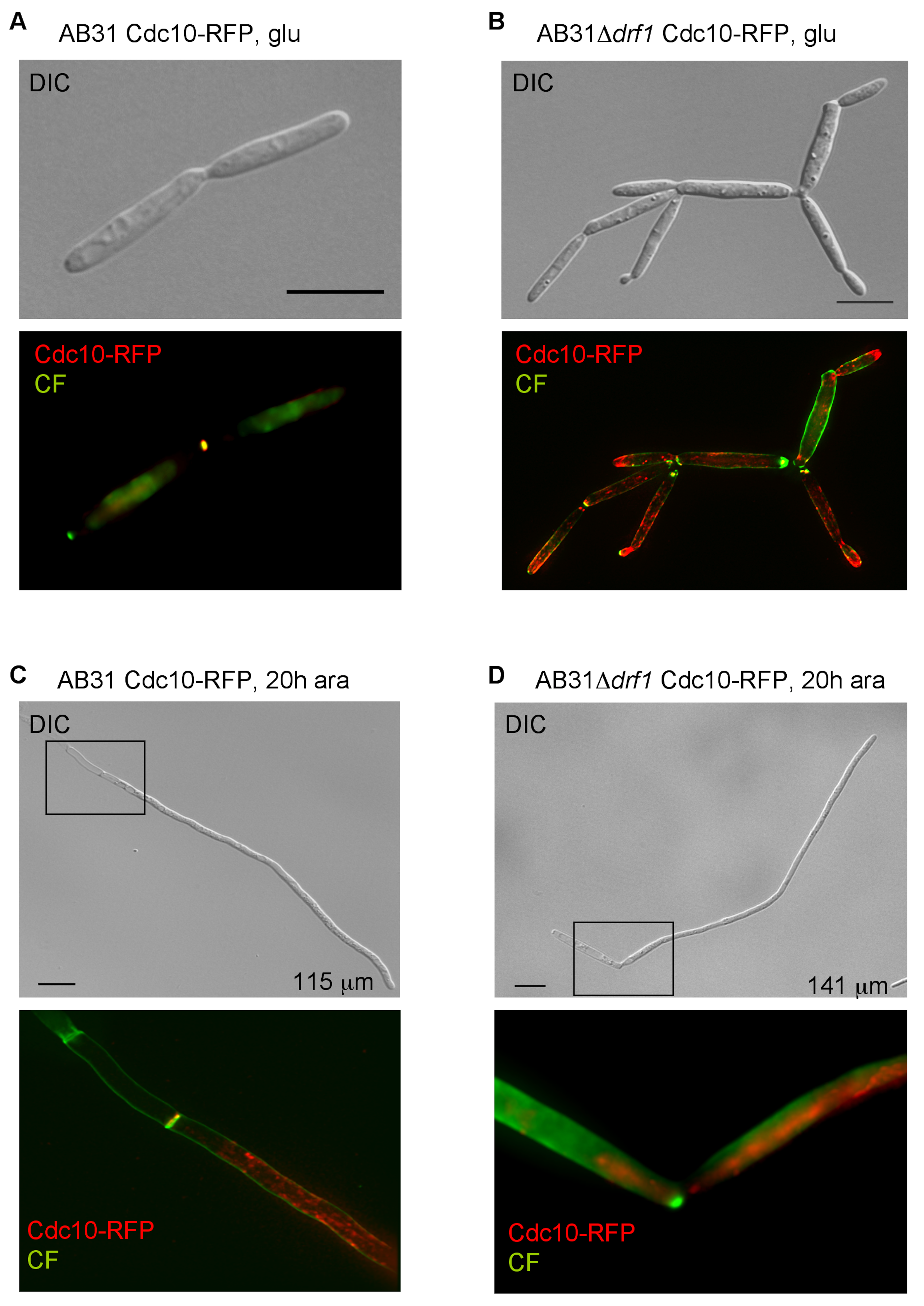

Supplement: Figure S8 — Septin rings depend on Drf1 during retraction septa formation. A: Cdc10-RFP is part of the septin ring during retraction septum formation in AB31 filaments independent of length. B: In short AB31Δdrf1 filaments septin collars are formed at the bud neck. C: Long AB31Δdrf1 filaments lack any specific septin structure. The length of each filament is measured using ImageJ and indicated at the bottom of the DIC image. (Scale bars: 10 µm) (TIFF) [file ppat.1002044.s008.tif]

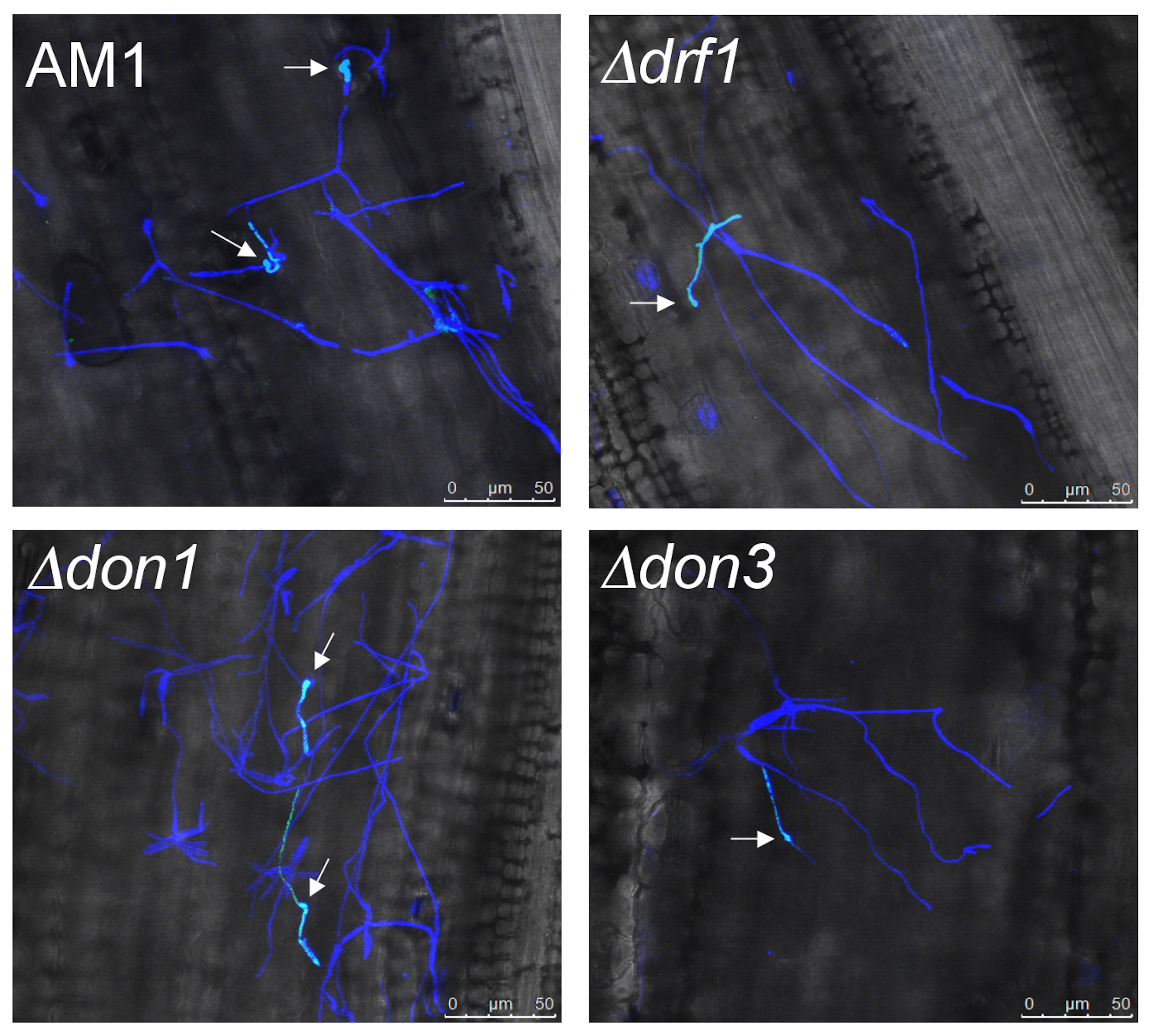

Supplement: Figure S9 — drf1, don1 and don3 mutants show reduced appressoria formation on the leaf surface. Seven-day-old maize seedlings were infected with SG200AM1 and the respective derivative strains. 20 h after infection the surface of the third leaf was analyzed by confocal microscopy. Fungal material was stained with calcofluor white (blue) and expression of the AM1 gfp-reporter (green) indicates appressorium formation (see arrows). The overlays of maximum projections of both channels with the corresponding bright-field image are depicted. (TIFF) [file ppat.1002044.s009.tif]

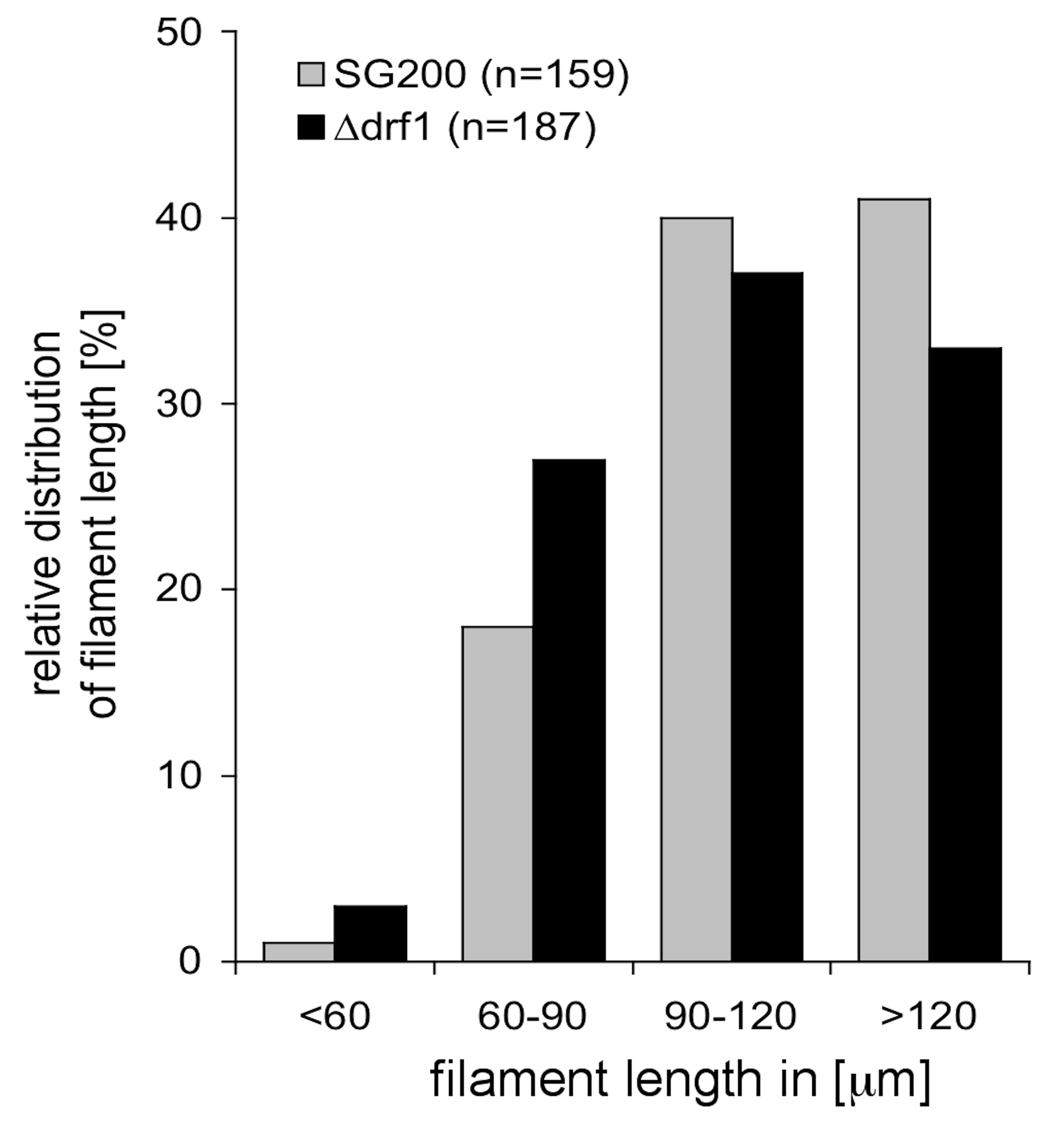

Supplement: Figure S10 — Filament length distribution is similar in SG200 AM1 and the drf1 mutant. The same images analyzed for figure 6 were used to measure the length of all filaments. (TIFF) [file ppat.1002044.s010.tif]

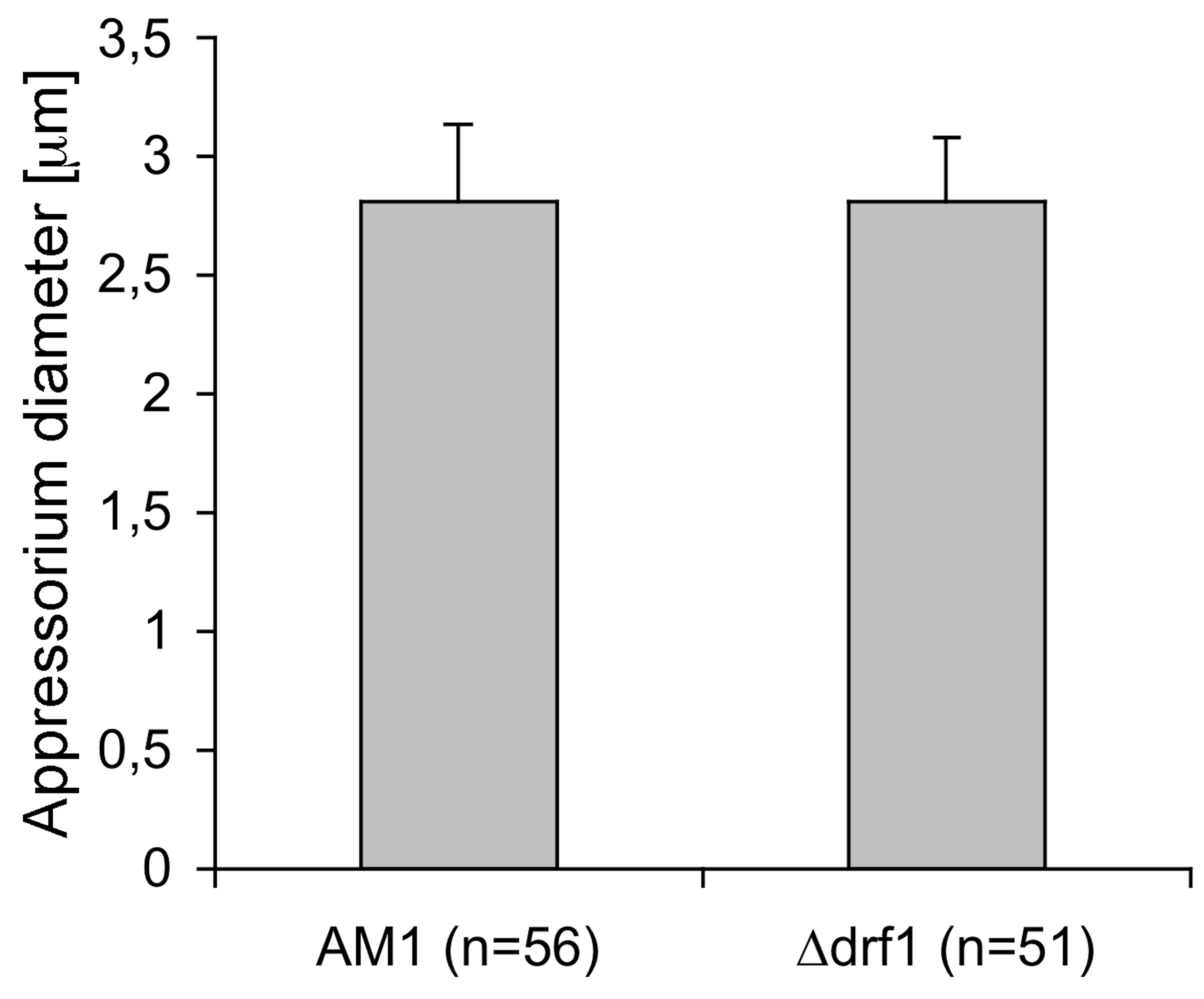

Supplement: Figure S11 — Appressoria diameter is similar in SG200 AM1 and the drf1 mutant. The same images analyzed for figure 6 were used to measure the diameter of the appressoria. Errorbars indicate standard deviation. (TIFF) [file ppat.1002044.s011.tif]
