# Supplementary material for: Septation of Infectious Hyphae Is Critical for Appressoria Formation and Virulence in the Smut Fungus Ustilago Maydis
Source: PLoS Pathog. 2011 May 19;7(5):e1002044. doi: 10.1371/journal.ppat.1002044 (PMC3098242; doi:10.1371/journal.ppat.1002044)
Supplement: Table S1 — U. maydis strains used in this study. (DOC) [file ppat.1002044.s012.doc]

**Table S1**

| Strain | Reference | Plasmid transformed | Integration locus | Progenitor strain |
| --- | --- | --- | --- | --- |
| Bub8 (*a2 b4*) | [21] |  |  |  |
| FB1 (*a1 b1*) | [21] |  |  |  |
| SG200AM1 (*a1::mfa2 bW2bE1*) | [12] |  |  |  |
| AB31 (*a2 Pcrg:bW2bE1*) | [58] |  |  |  |
| Bub8 P_etef_:GFP-Drf1 GBD | this study | pETEF-GFP-Drf1 GBD | *ip* | Bub8 |
| Bub8 P_etef_:GFP-Srf1 GBD | this study | pETEF-GFP-Srf1 GBD | *ip* | Bub8 |
| Bub8 ∆*drf1* | this study | p∆drf1-hyg | *drf1* | Bub8 |
| Bub8 ∆*drf1* P_etef_:drf1 | this study | pETEF-Drf1 | *ip* | Bub8 ∆drf1 |
| Bub8 ∆*drf1* P_etef_:drf1∆GBD | this study | pETEF-Drf1∆GBD | *ip* | Bub8 ∆drf1 |
| Bub8 ∆*cdc42* | [18] |  |  |  |
| Bub8 *don1* | [21] |  |  |  |
| Bub8 *don1* P_etef_:drf1 | this study | pETEF-Drf1 | *ip* | Bub8 ∆don1 |
| Bub8 *don1* P_etef_:drf1∆GBD | this study | pETEF-Drf1∆GBD | *ip* | Bub8 ∆don1 |
| FB1 ∆*drf1* | this study | p∆drf1-hyg | *drf1* | FB1 |
| AB31 P_cdc15_:cdc15-GFP | this study | pCond-Cdc15-GFP | *cdc15* | AB31 |
| AB31 ∆*drf1* P_cdc15_:cdc15-GFP | this study | p∆drf1-hyg | *drf1* | AB31 P_cdc15_:cdc15-GFP |
| AB31 ∆*don1* P_cdc15_:cdc15-GFP | this study | p∆don1-hyg | *don1* | AB31 P_cdc15_:cdc15-GFP |
| AB31 ∆*don3* P_cdc15_:cdc15-GFP, |  | p∆don3-hyg | *don3* |  |
| P_etef_:don3M^157A^ | this study | pETEF-Don3^M157A^ | *ip* | AB31 P_cdc15_:cdc15-GFP |
| AB31 P_etef_:Cdc10-RFP | this study | p123-Cdc10-RFP | *ip* | AB31 |
| AB31 ∆*drf1* P_etef_:Cdc10-RFP | this study | p123-Cdc10-RFP | *ip* | AB31 ∆drf1 |
| AB31 ∆*drf1* P_etef_:Drf1∆GBD-GFP | this study | pETEF-Drf1∆GBD-GFP | *ip* | AB31 ∆drf1 |
| AB31 P_etef_:Sec4-GFP | this study | pETEF-Sec4-GFP | *ip* | AB31 |
| AB31 ∆*drf1* P_etef_:Sec4-GFP | this study | pETEF-Sec4-GFP | *ip* | AB31 ∆drf1 |
| FB1 ∆*don1* | [21] |  |  |  |
| Bub8 ∆*don3* | [20] |  |  |  |
| FB1 ∆*don3* | [21] |  |  |  |
| FB1 ∆*cdc42* | [18] |  |  |  |
| SG200AM1 ∆*drf1* | this study | p∆drf1-hyg | *drf1* | SG200AM1 |
| SG200AM1 ∆*don1* | this study | p∆don1-hyg | *don1* | SG200AM1 |
| SG200AM1 ∆*don3* | this study | p∆don3-hyg | *don3* | SG200AM1 |
|  |  |  |  |  |
