# Supplementary material for: Septation of Infectious Hyphae Is Critical for Appressoria Formation and Virulence in the Smut Fungus Ustilago Maydis
Source: PLoS Pathog. 2011 May 19;7(5):e1002044. doi: 10.1371/journal.ppat.1002044 (PMC3098242; doi:10.1371/journal.ppat.1002044)
Supplement: Protocol S1 — Inhibition of the analog-sensitive Don3 kinase. (DOC) [file ppat.1002044.s013.doc]

**Supporting protocol:**

**Inhibition of the analog-sensitive Don3 kinase:** AB31∆*don3* cells expressing Petef:Don3^M157A^ and Cdc15-GFP for the studies of CAR formation in hyphae are grown to OD_600_=0.5 in glucose containing YEP medium. Filaments are induced by shifting the cells to arabinose containing YEP medium and Don3as is blocked by the addition of 1 µM NA-PP1 (Calbiochem) as described [36].
